# Efficient Synthesis of UDP-Furanoses via 4,5-Dicyanoimidazole(DCI)-Promoted Coupling of Furanosyl-1-Phosphates with Uridine Phosphoropiperidate

**DOI:** 10.3390/molecules24040655

**Published:** 2019-02-13

**Authors:** Wei-Jie Chen, Shuai-Bo Han, Zhen-Biao Xie, Hua-Shan Huang, Duo-Hua Jiang, Shan-Shan Gong, Qi Sun

**Affiliations:** Jiangxi Key Laboratory of Organic Chemistry, Jiangxi Science and Technology Normal University, 605 Fenglin Avenue, Nanchang 330013, China; chenweijie9@126.com (W.-J.C.); hanshuaibo1@126.com (S.-B.H.); xiezhenbiao1@126.com (Z.-B.X.); huanghuashan1@126.com (H.-S.H.); jiangduohua@126.com (D.-H.J.)

**Keywords:** NDP-sugar, P(V)-N activation, furanosyl-1-phosphate, nucleoside phosphoropiperidate, 4,5-dicyanoimidazole

## Abstract

A P(V)-N activation method based on nucleoside phosphoropiperidate/DCI system has been developed for improved synthesis of diverse UDP-furanoses. The reaction conditions including temperature, amount of activator, and reaction time were optimized to alleviate the degradation of UDP-furanoses to cyclic phosphates. In addition, an efficient and facile phosphoramidite route was employed for the preparation of furanosyl-1-phosphates.

## 1. Introduction

d-Galactofuranose (d-Gal*f*) is a ubiquitous glycosyl residue in the extracelluar glycans, glycoproteins, and glycosphingolipids of microorganisms, such as bacteria, protozoa, fungi, and parasites [1,2,3]. In addition, many other furanoses, such as d-glucofuranose (d-Glc*f*), d-fucofuranose (d-Fuc*f*), d-mannofuranose (d-Man*f*), l-arabinofuranose (l-Ara*f*), and l-fucofuranose (l-Fuc*f*) have also been found in various microbes and plants [4,5,6,7,8]. It has been revealed that furanose-containing polysaccharides and glycoconjugates are crucial for the survival and pathogenicity of many microorganisms, but completely absent in mammalian cells [9,10,11,12]. Therefore, their biosynthesis has long been recognized as a potential therapeutic target for microbial infections [13,14]. Currently, it has been elucidated that UDP-galactofuranose (UDP-Gal*f*) as the sugar donor is transformed from UDP-galactopyranose (UDP-Gal*p*) by UDP-Gal*p* mutase [15,16,17] and is the substrate of galactofuranosyltransferase for the incorporation into microbial sugar derivatives [18]. However, the limited availability of UDP-Gal*f* as well as other UDP-furanoses from enzymatic synthesis greatly hampers the identification of more UDP-pyranose mutases/furanosyltransferases and further understanding of their mechanism of actions [19].

Since the five-membered ring of furanoses is thermodynamically disfavored, furanosyl-1-phosphates are difficult to prepare [20]. Compared to UDP-pyranoses, UDP-furanoses are much more prone to degrade into furanosyl-1,2-cyclic phosphate and UMP even during the coupling process. Therefore, the chemical synthesis and purification of UDP-furanoses are notoriously inefficient [21]. Up to date, there are only very limited reports on the chemical synthesis of UDP-furanoses. The coupling of furanosyl-1-phosphate with activated UMPs, such as imidazolide [22,23,24], morpholidate [25,26], *N*-methyl imidazolide [27,28], has been utilized as the major approach to afford UDP-furanoses. The reactions of imidazolide and morpholidate are typically low-yielding (<20%) and require 1–2 days to finish. Although the employment of more reactive *N*-methyl imidazolide shortens the reaction time to a few hours, the yields of UDP-furanoses are low (8–35%). Alternatively, benzimidazolyl thiofuranosides have been prepared to couple with UDP [29]. The reaction rate of this method is fast (10 min–1 h). But the yields of UDP-furanoses are still within the range of 20–30%. In addition, the benzimidazolyl thiofuranosides have also been employed for the preparation of furanosyl-1-phosphates. The combined strength of chemical synthesis of furanosyl-1-phosphates and a galactose-1-phosphate uridylyltransferase provided a stereoselective chemoenzymatic approach to specific UDP-furanoses [30].

In our previous research, we developed a novel P(V)-N activation strategy based on nucleoside 5′-phosphoropiperidate/4,5-dicyanoimidazole (DCI) system for highly efficient synthesis of nucleoside 5′-polyphosphates [31,32,33]. Its further application on the synthesis of NDP-6-deoxy-l-pyranoses showed that piperidate/DCI system is much more reactive than the conventional imidazolide or morpholidate/1*H*-tetrazole system [34]. Herein, we report an improved synthesis of diverse UDP-furanoses based on the piperidate/DCI system. An efficient and facile phosphoramidite route for the preparation of furanosyl-1-phosphates was also developed.

## 2. Results and Discussion

### 2.1. A Phosphoramidite Approach for Furanosyl-1-Phosphate Synthesis

**Scheme 1:**
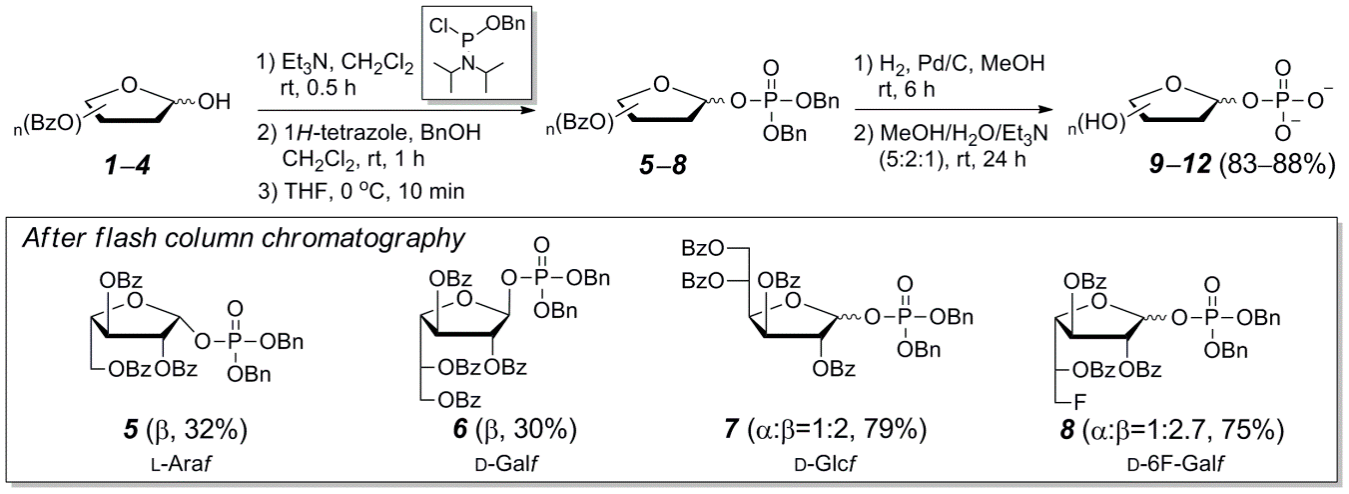
A phosphoramidite method for the synthesis of furanosyl-1-phosphates **9**–**12**.

For the P(V)–N activation strategy-based synthesis of UDP-furanoses, sugar-1-phosphate is an essential reactant but with poor availability. Previously, furanosyl-1-phosphates have been primarily prepared by coupling benzoyl-protected furanosyl-1-bromide with dibenzyl phosphate [20]. This method typically requires long reaction time and the yields are moderate (40–50%). Alternatively, furanosyl-1-phosphates have been prepared from thiofuranosides [30,35]. Based on our previous research on the phosphorylation of saccharides and nucleosides [36,37], we attempted to synthesize furanosyl-1-phosphates via a phosphoramidite route. As shown in Scheme 1, phosphitylation of 1-OH of Bz-protected furanoses **1**–**4** with benzyl-*N*,*N*-diisopropylchlorophosphoramidite, 1*H*-tetrazole- catalyzed alcoholysis, and in situ oxidation with H_2_O_2_ afforded crude **5**–**8** in good yields over three consecutive fast steps. However, we found that the α anomer of l-Ara*f* (**5**) decomposed completely during flash column chromatography and only the β anomer was isolated in 32% yield. Similarly, only the β anomer of d-Gal*f* (**6**) survived column purification and was obtained in 30% yield. In contrast, the phosphate triesters of d-Glc*f* (**7**) and d-6F-Gal*f* (**8**) were much more stable and isolated as anomeric mixtures in over 75% yields. Catalytic hydrogenation followed by treatment with MeOH/H_2_O/Et_3_N (5:2:1) gave the desired furanosyl-1-phosphates **9**–**12** in excellent yields.

### 2.2. The Attempt to Synthesize UDP-l-Araf (***16***) from Benzoyl-Protected l-Arabinofuranosyl-1-Phosphate (***13***)

**Figure 1:**
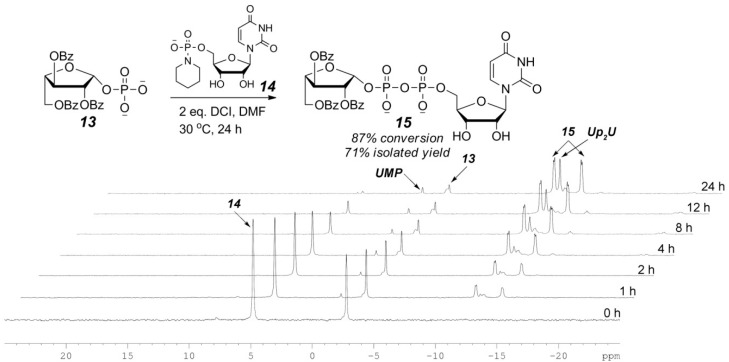
^31^P NMR tracing of the P(V)-N activation coupling reaction of **13** and **14**.

As mentioned above, UDP-furanoses are easy to decompose to 1,2-cyclic furanosyl phosphate due to the presence of free 2-OH. To avoid the formation of 1,2-cyclic phosphate byproduct, our initial attempt to synthesize UDP-furanoses via the P(V)-N activation strategy was to couple 2,3,5-tribenzoyl-protected l-Ara*f*-1-phosphate (**13**) with 2 equiv of uridine 5′-phosphoropiperidate (**14**) in the presence of 2 equiv of DCI in DMF at 30 °C. ^31^P NMR tracing experiment (Figure 1) showed that Bz-protected UDP-l-Ara*f* (**15**) formed smoothly over 24 h when piperidate **14** was completely consumed. While ~10% of the starting sugar phosphate **13** was left unreacted, its conversion to **15** was nearly 90% based on ^31^P NMR integration. Meanwhile, diuridine diphosphate (Up_2_U) and uridine monophosphate (UMP) were observed as the major byproducts. It is noteworthy that benzoyl-protection on l-Ara*f* moiety completely suppressed the formation of 1,2-cyclic phosphate byproduct and the desired **15** was isolated in 71% yield.

**Scheme 2:**
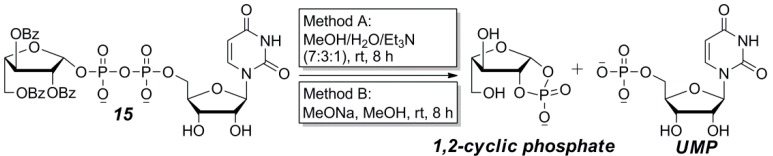
The decomposition of UDP-l-Araf (**15**) during benzoyl deprotection.

Although the P(V)-N activation strategy yielded an excellent result in the coupling step, we were also clearly aware of the possibility that the final deprotection step may cause undesired decomposition to sugar 1,2-cyclic phosphate. As shown in Scheme 2, both MeOH/H_2_O/Et_3_N (7:3:1) [38] and MeONa/MeOH [36] deprotection methods caused almost total breakdown of **15** to equal amounts of 1,2-cyclic phosphate and UMP, while the MeOH/TEAB buffer/Et_3_N (3:4:0.05) method reported by Williams et al. [39] was not effective at the claimed low temperature (−20 °C). The above experimental results reveal that the UDP-l-Ara*f* has very strong tendency to decompose via the intramolecular cyclization under basic conditions at room temperature.

### 2.3. The P(V)-N Activation Method for the Synthesis UDP-Furanoses from Furanosyl-1-Phosphates

**Figure 2:**
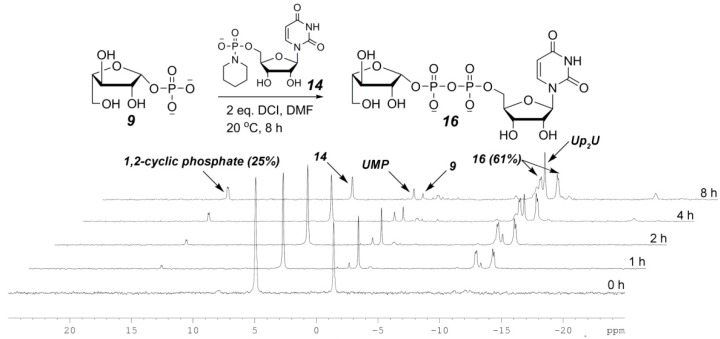
^31^P NMR tracing of the coupling reaction of **9** and **14** with 2 equiv of DCI.

The failure to deprotect **15** without causing decomposition urged us to go back to the direct coupling of phosphate **9** with piperidate **14** under the P(V)-N activation conditions (Figure 2). The reaction temperature was intentionally lowered to 20 °C to alleviate degradation of the UDP-l-Ara*f* product. As expected, the formation of 1,2-cyclic phosphate was observed. Surprisingly, the reaction between **14** and fully deprotected **9** was significantly faster (8 h) than that with Bz-protected **13**, which in turn reduced the undesired self-condensation of **14**. On the basis of ^31^P NMR integration, it was estimated that UDP-l-Ara*f* (**16**) and 1,2-cyclic phosphate were obtained in 61% and 25% yields, respectively.

**Figure 3:**
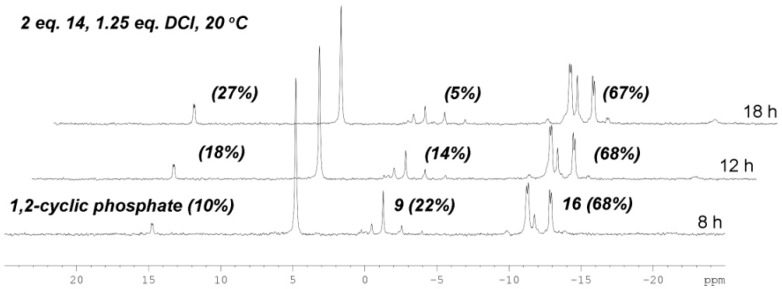
The composition of the coupling reaction of **9** and **14** with 1.25 equiv of DCI at different time points.

To suppress the degradation of **16** and self-condensation of **14**, we further reduced the amount of activating reagent. When 1.25 equiv of DCI was applied, close analysis of the composition of the reaction mixture at different reaction time showed that the yield of **16** reached the maximum level (68%) at 8 h when 22% of **9** remained unreacted and 10% 1,2-cyclic phosphate byproduct was formed (Figure 3). However, as the reaction proceeded, the yield of **16** did not increase anymore due to its degradation, which occurred in a comparable rate to its formation. When most of **9** was consumed (5% left) at 18 h, the formation of 1,2-cyclic phosphate (increased to 27%) and Up_2_U byproduct was further aggravated. Therefore, quenching the reaction at 8 h should be beneficial for easier purification.

**Scheme 3:**
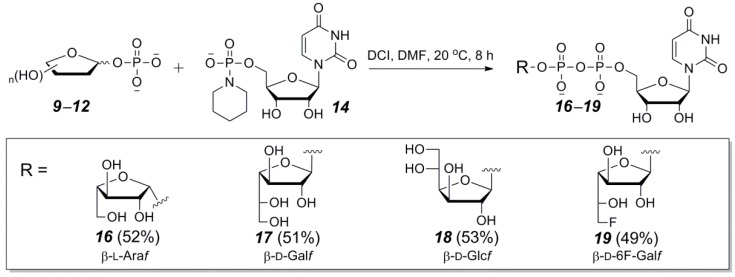
The P(V)-N activation method for the synthesis of UDP-furanoses (**16**–**19**).

With the optimized conditions, four UDP-furanoses **16**–**19** were prepared via the P(V)-N activation method. The coupling of furanosyl-1-phosphates **9**–**12** with 2 equiv of piperidate **14** in the presence of 1.25 equiv of DCI at 20 °C for 8 h afforded **16**–**19** in 49–53% yields after preparative HPLC purification (Scheme 3). It was determined that adjustment of the pH of TEAB buffer (10 mM) to 8.0 by adding acetic acid is helpful to minimize the decomposition of UDP-furanoses during HPLC purification. It is worth mentioning that ^31^P NMR tracing of the reactions of **18** and **19** showed that the anomeric configuration of furanosyl is an important factor for not only the stability of the UDP-furanose product but also the reactivity of the sugar-1-phosphate reactant. For instance, the UDP-α-d-Glc*f* was much labile than UDP-β-d-Glc*f* and completely decomposed during the reaction. When the reaction of d-6F-Gal*f*-1-phosphate (**12**) was stopped at 8 h, 95% of the β anomer had been converted to the product, but only 68% of the α anomer had reacted. After preparative HPLC purification, only UDP-β-d-6F-Gal*f* (**19**) was obtained as the sole product.

## 3. Materials and Methods 

### 3.1. General Methods

General chemical reagents and solvents were obtained from commercial suppliers. The Bz-protected furanoses (**1**–**4**) were prepared according to known procedures [27,29,40,41,42]. Uridine 5′-phosphoropiperidate (**14**) was prepared according to a previous report [32]. All reactions were performed under an atmosphere of inert gas and monitored by thin layer chromatography on plates coated with 0.25 mm silica gel 60 F254. TLC plates were visualized by UV irradiation (254 nm). Flash column chromatography employed silica gel (particle size 32–63 μm). All NMR spectra were obtained with a Bruker AV-400 instrument (Billerica, MA, USA) with chemical shifts reported in parts per million (ppm, *δ*) and referenced to CDCl_3_, MeOH-*d*_4_, or D_2_O. The NMR spectra of compounds **5**–**13** and **15**–**19** were provided in Supplementary Materials (Figures S1–S42). Low- and high-resolution mass spectra were reported as *m*/*z* and obtained with a Bruker amaZon SL and a Bruker Dalton microTOFQ II mass spectrometer, respectively. The HPLC traces of **16**–**19** were recorded on an Agilent 1260 instrument equipped with a Waters XTerra MS C18 analytical column (4.6 × 150 mm, 5 μm) [flow rate = 1.0 mL/min; linear gradient of 5% to 100% MeOH in TEAB buffer (10 mM, pH 8.0) over 10 min; UV detection at 254 nm] and provided in Supplementary Materials (Figures S43–S46).

### 3.2. General Synthetic Procedure and Characterization of Protected Furanosyl-1-Phosphates ***5***–***8***

To a solution of Bz-protected furanoses **1**–**4** (1.0 mmol) and Et_3_N (2.0 mmol) in CH_2_Cl_2_ (1.7 mL) was added a solution of benzyl-*N*,*N*-diisopropylchlorophosphoramidite (1.7 mmol) in CH_2_Cl_2_ (0.3 mL), dropwise, at 35 °C. The reaction was stirred for 30 min and concentrated in vacuo. The residue was co-evaporated with CH_3_CN (5 mL × 2), and redissolved in EtOAc (5 mL). The Et_3_N∙HCl salt was removed by filtration and the filtrate was concentrated to afford the crude phosphoramidite intermediate. To a solution of the intermediate in CH_2_Cl_2_ (10 mL) was added 1*H*-tetrazole (4.0 mmol) and benzyl alcohol (2 mmol). The reaction was stirred for 1 h, diluted with CH_2_Cl_2_ (20 mL), and washed with HCl aqueous solution (0.1 M, 10 mL) followed by deionized H_2_O (10 mL). The organic phase was dried over anhydrous Na_2_SO_4_ and concentrated in vacuo to give the crude phosphite triester intermediates, which were briefly purified by an ultrafast flash column chromatography method (PE/EA = 5:1, elution time = ~5 min). To an ice-cooled solution of the phosphite triesters in THF (10 mL) was added 30% H_2_O_2_ (10 mmol) dropwise. The reaction was stirred for 10 min and concentrated in vacuo. The residue was dissolved in EtOAc (30 mL) and the organic phase was washed with HCl aqueous solution (0.1 M, 10 mL) followed by deionized H_2_O (10 mL), dried over anhydrous Na_2_SO_4_, and concentrated in vacuo. Flash column chromatography (PE/EA = 6:1) afforded **5**–**8** in pure form.

*Dibenzyl 2,3,5-tri-O-benzoyl-β-l-arabinofuranosyl-1-phosphate* (**5**). The reaction of **1** (462 mg, 1.0 mmol) afforded **5** (231 mg, 32%) as a colorless syrup. ^1^H NMR (400 MHz, CDCl_3_) δ 8.08–8.03 (m, 6H), 7.62–7.55 (m, 2H), 7.52–7.44 (m, 3H), 7.39 (t, *J* = 7.7 Hz, 2H), 7.32 (t, *J* = 7.7 Hz, 3H), 7.28–7.24 (m, 5H), 7.21–7.18 (m, 2H), 7.12–7.09 (m, 2H), 6.34 (dd, *J*_1_ = 5.3 Hz, *J*_2_ = 4.6 Hz, 1H), 6.05–5.99 (m, 1H), 5.77–5.72 (m, 1H), 5.06–4.99 (m, 1H), 4.98–4.89 (m, 2H), 4.88–4.81 (m, 1H), 4.80–4.74 (m, 1H), 4.68–4.59 (m, 2H) ppm; ^13^C NMR (100 MHz, CDCl_3_) δ 166.0, 165.7, 165.5, 135.4, 133.7, 133.6, 133.1, 130.0, 129.9, 129.8, 129.6, 128.8, 128.4, 128.3, 127.7, 127.6, 98.1, 80.0, 75.1, 69.3, 65.3 ppm; ^31^P NMR (162 MHz, CDCl_3_) δ −2.4 ppm; LRMS (ESI+) *m*/*z* calcd for C_40_H_36_O_11_P^+^ [M + H]^+^ 723.2; found 723.2.

*Dibenzyl 2,3,5,6-tetra-O-benzoyl-β-d-galactofuranosyl-1-phosphate* (**6**). The reaction of **2** (596 mg, 1.0 mmol) afforded **6** (257 mg, 30%) as a colorless syrup. ^1^H NMR (400 MHz, CDCl_3_) δ 8.03 (d, *J* = 7.8 Hz, 4H), 7.93 (d, *J* = 7.6 Hz, 2H), 7.85 (d, *J* = 7.9 Hz, 2H), 7.60 (t, *J* = 7.4 Hz, 1H), 7.56–7.47 (m, 3H), 7.42 (t, *J* = 7.6 Hz, 2H), 7.38–7.24 (m, 15H), 6.13 (d, *J* = 4.8 Hz, 1H), 6.08–6.05 (m, 1H), 5.63 (d, *J* = 4.3 Hz, 1H), 5.53 (s, 1H), 5.18–5.09 (m, 4H), 4.72 (t, *J* = 3.9 Hz, 1H), 4.65 (d, *J* = 5.6 Hz, 2H) ppm; ^13^C NMR (100 MHz, CDCl_3_) δ 164.8, 164.5, 164.4, 164.1, 134.5, 134.4, 134.3, 132.6, 132.5, 132.2 132.0, 129.0, 128.8, 128.7, 128.4, 127.7, 127.6, 127.5, 127.4, 127.3, 127.0, 126.8, 102.1, 82.9, 81.0, 76.2, 69.1, 68.6, 68.5, 62.4 ppm; ^31^P NMR (162 MHz, CDCl_3_) δ −3.0 ppm; LRMS (ESI+) *m*/*z* calcd for C_48_H_42_O_13_P^+^ [M + H]^+^ 857.2; found 857.2.

*Dibenzyl 2,3,5,6-tetra-O-benzoyl-d-glucofuranosyl-1-phosphate* (**7**). The reaction of **3** (596 mg, 1.0 mmol) afforded **7** (676 mg, 79%) as a colorless syrup. α-Isomer: ^1^H NMR (400 MHz, CDCl_3_) δ 8.06 (d, *J* = 7.6 Hz, 2H), 7.97 (d, *J* = 8.0 Hz, 2H), 7.83 (d, *J* = 7.7 Hz, 2H), 7.75 (d, *J* = 6.6 Hz, 2H), 7.64 (t, *J* = 7.4 Hz, 2H), 7.45–7.24 (m, 15H), 7.24–7.19 (m, 2H), 7.12 (d, *J* = 6.6 Hz, 2H), 6.38 (t, *J* = 4.8 Hz, 1H), 6.23 (t, *J* = 5.8 Hz, 1H), 5.81–5.78 (m, 2H), 5.62 (s, 1H), 5.08–5.01 (m, 2H), 4.98–4.94 (m, 3H), 4.89–4.85 (m, 1H), 4.60 (dd, *J*_1_ = 5.2, *J*_2_ = 12.3 Hz, 1H) ppm; ^13^C NMR (100 MHz, CDCl_3_) δ 166.0, 165.4, 165.2, 165.0, 135.4, 133.7, 133.5, 133.2, 133.1, 130.0, 129.9, 129.7, 129.6, 128.6, 128.5, 128.4, 128.3, 128.2, 127.8, 127.7, 97.4, 77.7, 75.3, 73.8, 69.4, 69.2, 63.3 ppm; ^31^P NMR (162 MHz, CDCl_3_) δ −2.6 ppm; β-Isomer: ^1^H NMR (400 MHz, CDCl_3_) δ 8.08 (d, *J* = 7.6 Hz, 2H), 8.01 (d, *J* = 7.6 Hz, 2H), 7.91 (d, *J* = 7.6 Hz, 2H), 7.77 (d, *J* = 7.3 Hz, 2H), 7.57 (t, *J* = 7.3 Hz, 2H), 7.56–7.45 (m, 5H), 7.45–7.24 (m, 13H), 6.19 (d, *J* = 4.8 Hz, 1H), 6.04 (d, *J* = 5.1 Hz, 1H), 5.97–5.95 (m, 1H), 5.61 (s, 1H), 5.34–5.25 (m, 2H), 5.22–5.18 (m, 3H), 4.99–4.96 (m, 3H), 4.72–4.67 (m, 1H) ppm; ^13^C NMR (100 MHz, CDCl_3_) δ 166.0, 164.9, 164.7, 164.6, 135.5, 135.4, 135.3, 133.9, 133.5, 133.1, 133.0, 130.0, 129.9, 129.7, 129.5, 128.7, 128.6, 128.5, 128.4, 128.3, 128.2, 128.0, 127.9, 102.7, 80.7, 80.4, 73.4, 69.7, 69.6, 69.5, 63.6 ppm; ^31^P NMR (162 MHz, CDCl_3_) δ −3.2 ppm; LRMS (ESI+) *m*/*z* calcd for C_48_H_42_O_13_P^+^ [M + H]^+^ 857.2; found 857.2.

*Dibenzyl 2,3,5-tri-O-benzoyl-6-deoxy-6-fluoro-d-galactofuranosyl-1-phosphate* (**8**). The reaction of **4** (494 mg, 1.0 mmol) afforded **8** (565 mg, 75%) as a colorless syrup. α-Isomer: ^1^H NMR (400 MHz, CDCl_3_) δ 8.28 (d, *J* = 7.6 Hz, 2H), 8.13 (d, *J* = 8.2 Hz, 4H), 7.73 (t, *J* = 7.4 Hz, 2H), 7.69–7.61 (m, 2H), 7.57 (t, *J* = 7.5 Hz, 2H), 7.53–7.37 (m, 9H), 7.29 (d, *J* = 6.6 Hz, 2H), 7.21 (d, *J* = 7.0 Hz, 2H), 6.47 (t, *J* = 5.0 Hz, 1H), 6.29 (d, *J* = 8.0 Hz, 2H), 6.07–5.95(m, 1H), 5.88–5.82 (m, 2H), 5.78 (dd, *J* = 16.5, 4.7 Hz, 1H), 5.15–5.04 (m, 1H), 4.75–4.69 (m, 3H) ppm; ^13^C NMR (100 MHz, CDCl_3_) δ 165.5, 165.1, 135.6, 135.5, 135.4, 130.1, 129.9, 129.8, 128.6, 128.5, 128.4, 127.8, 97.6, 80.3 (d, *J* = 172 Hz), 79.1, 76.5, 71.2, 69.5, 69.3 ppm; ^31^P NMR (162 MHz, CDCl_3_) δ −2.8 ppm; β-Isomer: ^1^H NMR (400 MHz, CDCl_3_) δ 8.17 (d, *J* = 7.5 Hz, 4H), 8.00 (d, *J* = 7.6 Hz, 2H), 7.73 (t, *J* = 7.4 Hz, 1H), 7.69–7.61 (m, 2H), 7.57 (t, *J* = 7.6 Hz, 2H), 7.53–7.37 (m, 9H), 6.26 (d, *J* = 4.6 Hz, 1H), 6.04–5.94 (m, 1H), 5.72 (d, *J* = 4.5 Hz, 1H), 5.56 (s, 1H), 5.32–5.23 (m, 4H), 4.99–4.91 (m, 1H), 4.84–4.79 (m, 2H) ppm; ^13^C NMR (100 MHz, CDCl_3_) δ 165.6, 165.1, 133.7, 133.6, 133.4, 130.1, 130.0, 129.9, 128.6, 128.5, 128.4, 128.0, 127.9, 103.1, 83.0, 82.1, 80.9 (d, *J* = 173 Hz), 76.9, 70.3, 70.1, 69.7, 69.5 ppm; ^31^P NMR (162 MHz, CDCl_3_) δ −3.5 ppm; LRMS (ESI+) *m*/*z* calcd for C_41_H_37_PO_11_F^+^ [M + H]^+^ 755.2; found 755.2.

### 3.3. General Synthetic Procedure and Characterization of Furanosyl-1-Phosphates ***9***–***13***

To a solution of phosphite triesters **5**–**8** (200 mg) and 2 equiv of Et_3_N in dry methanol (3 mL) was added 5wt% Pd/C (20 mg). The reaction was stirred under an atmosphere of hydrogen at ambient temperature for 8 h. Then, the catalyst was filtered off. The filtrate was dried to afford crude phosphate. For compound **13**, the crude product was directly dissolved in CH_2_Cl_2_ and purified by Sephadex LH-20 size exclusion gel chromatography. For compounds 9–12, the crude products were dissolved in MeOH/H_2_O/Et_3_N (5:2:1, 10 mL) and stirred for 24 h at ambient temperature. After concentration, the crude product was dissolved in MeOH and purified by Sephadex LH-20 size exclusion gel chromatography. A combination of appropriate fractions and concentration afforded **9**–**13** in triethylammonium salt form.

*Triethylammonium β-l-arabinofuranosyl-1-phosphate* (**9**). The reaction of **5** (200 mg, 0.28 mmol) afforded **9** (73 mg, 83%) as a colorless syrup. ^1^H NMR (400 MHz, D_2_O) δ 5.54 (d, *J* = 3.6 Hz, 1H), 4.16 (d, *J* = 4.4 Hz, 2H), 3.93–3.89 (m, 1H), 3.81 (dd, *J*_1_ = 12.5 Hz, *J*_2_ = 2.8 Hz, 1H), 3.69 (dd, *J*_1_ = 12.5 Hz, *J*_2_ = 5.8 Hz, 1H), 3.21 (q, *J* = 7.6 Hz, 6H), 1.29 (t, *J* = 7.6 Hz, 9H) ppm; ^13^C NMR (100 MHz, D_2_O) δ 97.1, 82.6, 76.9, 73.6, 62.4, 47.0, 8.5 ppm; ^31^P NMR (162 MHz, D_2_O) δ −0.8 ppm; LRMS (ESI−) *m*/*z* calcd for C_5_H_10_O_8_P^–^ [M − H]^−^ 229.1; found 229.1.

*Triethylammonium β-d-galactofuranosyl-1-phosphate* (**10**). The reaction of **6** (200 mg, 0.23 mmol) afforded **10** (83 mg, 85%) as a colorless syrup. ^1^H NMR (400 MHz, D_2_O) δ 5.38 (d, *J* = 6.1 Hz, 1H), 4.07 (s, 1H), 4.01 (dd, *J*_1_ = 6.0 Hz, *J*_2_ = 4.2 Hz, 2H), 3.97 (dd, *J*_1_ = 5.7 Hz, *J*_2_ = 3.2 Hz, 1H), 3.75–3.68 (m, 1H), 3.63 (dd, *J*_1_ = 11.6 Hz, *J*_2_ = 4.6 Hz, 1H), 3.55 (dd, *J*_1_ = 11.6 Hz, *J*_2_ = 7.2 Hz, 1H), 3.22 (q, *J* = 7.2 Hz, 6H), 1.23 (t, *J* = 7.2 Hz, 9H) ppm; ^13^C NMR (100 MHz, D_2_O): δ 102.5, 83.2, 81.6, 76.4, 70.5, 62.2, 46.2, 7.8 ppm; ^31^P NMR (162 MHz, D_2_O) δ −0.3 ppm; LRMS (ESI−) *m*/*z* calcd for C_6_H_12_O_9_P^–^ [M − H]^−^ 259.0; found 259.0.

*Triethylammonium d-glucofuranosyl-1-phosphate* (**11**). The reaction of **7** (200 mg, 0.23 mmol) afforded **11** (90 mg, 88%) as a colorless syrup. α-Isomer: ^1^H NMR (400 MHz, D_2_O) δ 5.57 (t, *J* = 5.2 Hz, 1H), 4.24 (t, *J* = 4.0 Hz, 1H), 4.15–4.10 (m, 2H), 3.81–3.86 (m, 1H), 3.75–3.66 (m, 2H), 3.23 (q, *J* = 7.2 Hz, 6H), 1.25 (t, *J* = 7.2 Hz, 9H) ppm; ^13^C NMR (100 MHz, D_2_O) δ 97.8, 80.7 78.3, 77.3, 75.2, 63.1, 46.8, 8.2 ppm; ^31^P NMR (162 MHz, D_2_O) δ −0.9 ppm; β-Isomer: ^1^H NMR (400 MHz, D_2_O) δ 5.37 (d, *J* = 6.3 Hz, 1H), 4.19–4.14 (m, 2H), 4.12–4.04 (m, 1H), 3.98–3.90 (m, 1H), 3.77–3.65 (m, 2H), 2.93 (q, *J* = 7.2 Hz, 6H), 1.16 (t, *J* = 7.2 Hz, 9H) ppm; ^13^C NMR (100 MHz, D_2_O) δ 103.4, 82.0, 80.7, 75.0, 69.7, 63.5, 46.9, 8.3 ppm; ^31^P NMR (162 MHz, D_2_O) δ −1.9 ppm; LRMS (ESI−) *m*/*z* calcd for C_6_H_12_O_9_P^–^ [M − H]^−^ 259.0; found 259.0.

*Triethylammonium 6-deoxy-6-fluoro-d-galactofuranosyl-1-phosphate* (**12**). The reaction of **8** (200 mg, 0.27 mmol) afforded **12** (112 mg, 86%) as a colorless syrup. α-Isomer: ^1^H NMR (400 MHz, D_2_O) δ 5.55 (d, *J* = 4.7 Hz, 1H), 4.74–4.48 (m, 2H), 4.31–3.88 (m, 4H), 2.93 (q, *J* = 7.2 Hz, 6H), 1.19 (t, *J* = 7.2 Hz, 9H) ppm; ^13^C NMR (100 MHz, D_2_O) δ 96.5, 83.6 (d, *J* = 162 Hz), 80.5, 76.2, 73.2, 70.1 46.3, 7.9 ppm; ^31^P NMR (162 MHz, D_2_O) δ −0.76 ppm; β-Isomer: ^1^H NMR (400 MHz, D_2_O) δ 5.53 (d, *J* = 5.8 Hz, 1H), 4.74–4.48 (m, 2H), 4.31–3.88 (m, 4H), 2.93 (q, *J* = 7.2 Hz, 6H), 1.19 (t, *J* = 7.2 Hz, 9H) ppm; ^13^C NMR (100 MHz, D_2_O) δ 102.9, 84.2(d, *J* = 165 Hz), 83.0, 81.5, 76.4, 69.1, 46.3, 7.9 ppm; ^31^P NMR (162 MHz, D_2_O) δ −1.8 ppm; LRMS (ESI−) *m*/*z* calcd for C_6_H_11_PO_8_F^–^ [M − H]^−^ 261.1; found 261.1.

*Triethylammonium 2,3,5-tri-O-benzoyl-β-l-arabinofuranosyl-1-phosphate* (**13**). The reaction of **5** (200 mg, 0.28 mmol) afforded **13** (179 mg, 98%) as a colorless syrup. ^1^H NMR (400 MHz, CDCl_3_) δ 8.11 (d, *J* = 7.7 Hz, 2H), 7.98 (dd, *J*_1_ = 11.6 Hz, *J*_2_ = 8.0 Hz, 2H), 7.58–7.43 (m, 3H), 7.43–7.32 (m, 5H), 7.27 (t, *J* = 7.6 Hz, 2H), 6.22–6.10 (m, 1H), 6.04 (t, *J* = 6.2 Hz, 1H), 5.73–5.63 (m, 1H), 4.75 (d, *J* = 6.3 Hz, 2H), 4.50 (q, *J* = 6.0 Hz, 1H), 2.91 (q, *J* = 8.0 Hz, 6H), 1.14 (t, *J* = 8.0 Hz, 9H) ppm; ^13^C NMR (100 MHz, CDCl_3_) δ 166.3, 166.0, 165.8, 133.4, 133.1, 132.9, 130.3, 129.9 (×2), 129.5, 128.5, 128.4, 128.3, 96.6, 78.6, 77.6, 77.3, 77.1, 77.0, 66.6, 45.4, 8.6 ppm; ^31^P NMR (162 MHz, CDCl_3_) δ −0.8 ppm; LRMS (ESI−) *m*/*z* calcd for C_26_H_22_O_11_P^–^ [M − H]^−^ 541.1; found 541.1.

### 3.4. General Synthetic Procedure and Characterization of UDP-Furanoses ***15***–***19***

To a solution of uridine 5′-phosphoropiperidate (0.4 mmol) and furanosyl-1-phosphates **9**–**13** (0.2 mmol) in dry DMF was added 4,5-dicyanoimidazole (DCI, 0.25 mmol (**9**–**12**) or 0.4 mmol (**13**)). The reaction was stirred at 20 °C for 8 h (**9**–**12**) or 30 °C for 24 h (**13**). Then, the solution was concentrated in vacuo. The residues of **16**–**19** was dissolved in TEAB buffer (0.5 mL, pH = 8.0) and purified by a preparative reverse phase HPLC (XTerra Prep MS C18, 10 μm, 19 × 250 mm) [flow rate = 20 mL/min; linear gradient of 5% to 15% MeOH in TEAB buffer (10 mM, pH 8.0) over 15 min; UV detection at 254 nm; the retention time of **16**–**19** was between 5–7 min]. Compound **15** was purified using NH_4_HCO_3_ buffer (10 mM, pH = 8.5) [flow rate = 20 mL/min; linear gradient of 10% to 55% MeOH in NH_4_HCO_3_ buffer over 5 min, then 55% to 100% MeOH in NH_4_HCO_3_ buffer over 10 min; UV detection at 254 nm; the retention time of **15** was 12.8 min]. Combination of appropriate fractions and lyophilization afforded products **16**–**19** in triethylammonium salt form. The ammonium salt of **15** was further converted into disodium salt by passing through a small column of Na^+^ ion exchange resin.

*UDP-2,3,5-tri-O-benzoyl-β-l-arabinofuranose* (**15**). The reaction of **13** (20 mg, 0.031 mmol) afforded **15** (20 mg, 71%) as disodium salt, a glassy solid. ^1^H NMR (400 MHz, MeOH-*d*_4_) δ 8.11 (d, *J* = 7.5 Hz, 2H), 7.99 (d, *J* = 7.3 Hz, 3H), 7.94 (d, *J* = 7.3 Hz, 2H), 7.64–7.54 (m, 2H), 7.54–7.41 (m, 5H), 7.31 (t, *J* = 7.8 Hz, 2H), 6.21 (s, 1H), 6.01 (t, *J* = 6.3 Hz, 1H), 5.92 (d, *J* = 5.0 Hz, 1H), 5.80 (d, *J* = 8.0 Hz, 1H), 5.75–5.66 (m, 1H), 4.75 (d, *J* = 6.3 Hz, 2H), 4.58 (q, *J* = 6.0 Hz, 1H), 4.29 (t, *J* = 4.4 Hz, 1H), 4.24–4.14 (m, 3H), 4.05 (s, 1H) ppm; ^13^C NMR (100 MHz, MeOH-*d*_4_) δ 165.2, 164.7, 164.6, 163.8, 150.2, 140.3, 132.1, 132.0, 131.7, 128.7, 128.5, 128.3, 128.2, 128.1, 127.2, 127.1, 127.0, 100.8, 95.9, 87.4, 82.5, 77.1, 75.7, 73.2, 68.8, 64.8, 63.7 ppm; ^31^P NMR (162 MHz, MeOH-*d*_4_) δ −10.3, −12.7 ppm; IR *ν*_max_ 3563, 3062, 2975, 2927, 2893, 1723, 1698, 1682, 1602, 1585, 1452, 1316, 1266, 1179, 1111, 1071, 1047, 1001, 936, 880, 709 cm^−1^; HRMS (ESI−) *m*/*z* calcd for C_35_H_33_N_2_O_19_P_2_^–^ [M − H]^−^ 847.1158; found 847.1153.

*UDP-β-l-arabinofuranose* (**16**). The reaction of **9** (20 mg, 0.063 mmol) afforded **16** (24 mg, 52%) as bis(triethylammonium) salt, a glassy solid. ^1^H NMR (400 MHz, D_2_O) δ 7.88 (d, *J* = 8.1 Hz, 1H), 5.88 (d, *J* = 8.4 Hz, 2H), 5.55 (s, 1H), 4.29 (s, 2H), 4.23–4.17 (m, 3H), 4.07 (d, *J* = 4.7 Hz, 2H), 3.83 (s, 1H), 3.72 (d, *J* = 12.5 Hz, 1H), 3.65–3.55 (m, 1H), 3.12 (q, *J* = 7.3 Hz, 12H), 1.19 (t, *J* = 7.3 Hz, 18H) ppm; ^13^C NMR (100 MHz, D_2_O) δ 167.7, 153.3, 143.1, 104.2, 99.3, 89.9, 84.8, 84.2, 78.2, 75.2, 74.9, 71.2, 66.5, 63.8, 48.2, 9.7 ppm; ^31^P NMR (162 MHz, D_2_O) δ −11.1, −12.4 ppm; LRMS (ESI−) *m*/*z* calcd for C_14_H_21_N_2_O_16_P_2_^–^ [M − H]^−^ 535.0; found 535.0.

*UDP-β-d-galactofuranose* (**17**). The reaction of 10 (20 mg, 0.048 mmol) afforded **17** (19 mg, 51%) as bis(triethylammonium) salt, a glassy solid. ^1^H NMR (400 MHz, D_2_O) δ 7.90 (d, *J* = 7.9 Hz, 1H), 5.91 (d, *J* = 9.6 Hz, 2H), 5.52 (d, *J* = 5.3 Hz, 1H), 4.31 (s, 2H), 4.22–4.10 (m, 5H), 4.02 (s, 1H), 3.76 (s, 1H), 3.71–3.52 (m, 2H), 3.13 (q, *J* = 7.2 Hz, 12H), 1.21 (t, *J* = 7.2 Hz, 18H) ppm; ^13^C NMR (100 MHz, D_2_O) δ 167.0, 152.6, 142.4, 104.7, 103.5, 89.1, 85.0, 84.1, 82.7, 77.6, 74.6, 71.6, 70.5, 65.7, 63.4, 47.5, 9.0 ppm; ^31^P NMR (162 MHz, D_2_O) δ −11.2, −13.5 ppm; LRMS (ESI−) *m*/*z* calcd for C_15_H_23_N_2_O_17_P_2_^–^ [M − H]^−^ 565.0; found 565.0.

*UDP-β-d-glucofuranose* (**18**). The reaction of **11** (20 mg, 0.046 mmol) afforded **18** (19 mg, 53%) as bis(triethylammonium) salt, a glassy solid. ^1^H NMR (400 MHz, D_2_O) δ 7.87 (d, *J* = 8.1 Hz, 1H), 5.89 (d, *J* = 7.8 Hz, 2H), 5.49 (d, *J* = 6.2 Hz, 1H), 4.37 (s, 2H), 4.29 (s, 1H), 4.24–4.17 (m, 5H), 4.00 (s, 1H), 3.79 (d, *J* = 11.8 Hz, 1H), 3.63 (dd, *J*_1_ = 12.0, *J*_2_ = 5.8 Hz, 1H), 3.11 (q, *J* = 7.2 Hz, 12H), 1.19 (t, *J* = 7.2 Hz, 18H) ppm; ^13^C NMR (100 MHz, D_2_O) δ 166.3, 152.0, 141.8, 104.0, 102.8, 88.5, 83.5, 82.3, 80.6, 75.0, 73.9, 69.9, 65.1, 64.2, 63. 6, 46.9, 8.4; ^31^P NMR (162 MHz, D_2_O) δ −11.1, −13.8 ppm; LRMS (ESI−) *m*/*z* calcd for C_15_H_23_N_2_O_17_P_2_^–^ [M − H]^−^ 565.0; found 565.0.

*UDP-6-deoxy-6-fluoro-β-d-galactofuranose* (**19**). The reaction of **12** (20 mg, 0.048 mmol) afforded **19** (18 mg, 49%) as bis(triethylammonium) salt, a glassy solid. ^1^H NMR (400 MHz, D_2_O) δ 8.00 (d, *J* = 8.1 Hz, 1H), 6.02 (d, *J* = 8.2 Hz, 2H), 5.65 (d, *J* = 5.5 Hz, 1H), 4.73–4.52 (m, 2H), 4.42 (s, 2H), 4.33–4.24 (m, 5H), 4.15–4.05 (m, 2H), 3.24 (q, *J* = 7.3 Hz, 12H), 1.32 (t, *J* = 7.3 Hz, 18H) ppm; ^13^C NMR (100 MHz, D_2_O) δ 166.4, 152.1, 141.9, 104.2, 103.0, 88.6, 84.8 (d, *J* = 173 Hz), 84.0, 83.6, 82.1, 77.0, 74.0, 70.0, 69.7, 65.2, 47.0, 8.5 ppm; ^31^P NMR (162 MHz, D_2_O) δ −10.8, −13.2 ppm; LRMS (ESI−) *m*/*z* calcd for C_15_H_22_FN_2_O_16_P_2_^–^ [M − H]^−^ 567.0; found 567.0.

## 4. Conclusions

In summary, our attempt to synthesize UDP-furanoses via the phosphoropiperidate/DCI system-based P(V)-N activation strategy afforded a more efficient approach. Due to the labile nature of UDP-furanoses, reaction conditions such as temperature (20 °C), amount of activator (1.25 equiv), and reaction time (8 h) were optimized to alleviate the degradation of UDP-furanoses to sugar 1,2-cyclic phosphates. In addition, a phosphoramidite approach for the preparation of furanosyl-1-phosphates, which involves three consecutive fast steps, was also developed. Although the deprotection of benzoyl groups of UDP-l-Ara*f* precursor **15** caused severe degradation to l-Ara*f*-1,2-cyclic phosphate, the much higher coupling efficacy of protected sugar-1-phosphate and complete absence of product degradation exhibited a huge advantage over the coupling with fully deprotected furanosyl-1-phosphates. Future discovery of an efficient and selective deprotection method for benzoyl groups could greatly improve the chemical synthesis of UDP-furanoses.

## Supplementary Materials

The following are available online. Figure S1–S42: The NMR spectra of compounds **5**–**13** and **15**–**19**; Figure S43–S46: The HPLC traces of **16**–**19**.
