# Supplementary material for: Efficient Synthesis of UDP-Furanoses via 4,5-Dicyanoimidazole(DCI)-Promoted Coupling of Furanosyl-1-Phosphates with Uridine Phosphoropiperidate
Source: Molecules. 2019 Feb 13;24(4):655. doi: 10.3390/molecules24040655 (PMC6412210; doi:10.3390/molecules24040655)
Supplement: Supplementary file 1 [file molecules-24-00655-s001.pdf]

Supporting Information

Efficient synthesis of UDP-furanoses via 4,5-dicyanoimidazole(DCI)-promoted coupling of furanosyl-1-phosphates with uridine phosphoropiperidate

Wei-Jie Chen, Shuai-Bo Han, Zhen-Biao Xie, Hua-Shan Huang, Duo-Hua Jiang, Shan-Shan Gong* and Qi Sun*

Jiangxi Key Laboratory of Organic Chemistry, Jiangxi Science and Technology Normal University, 605 Fenglin Avenue, Nanchang, Jiangxi 330013, PR China

E-mails: gongshanshan@jxstnu.edu.cn;sunqi@jxstnu.edu.cn

Table of contents

1. The NMR spectra of **5**–**8**	Page S2–S7
2. The NMR spectra of **9**–**13**	Page S8–S15
3. The NMR spectra of **15**–**19**	Page S15–S22
4. The HPLC traces of **16**–**19**	Page S23

1. The NMR spectra of 5–8

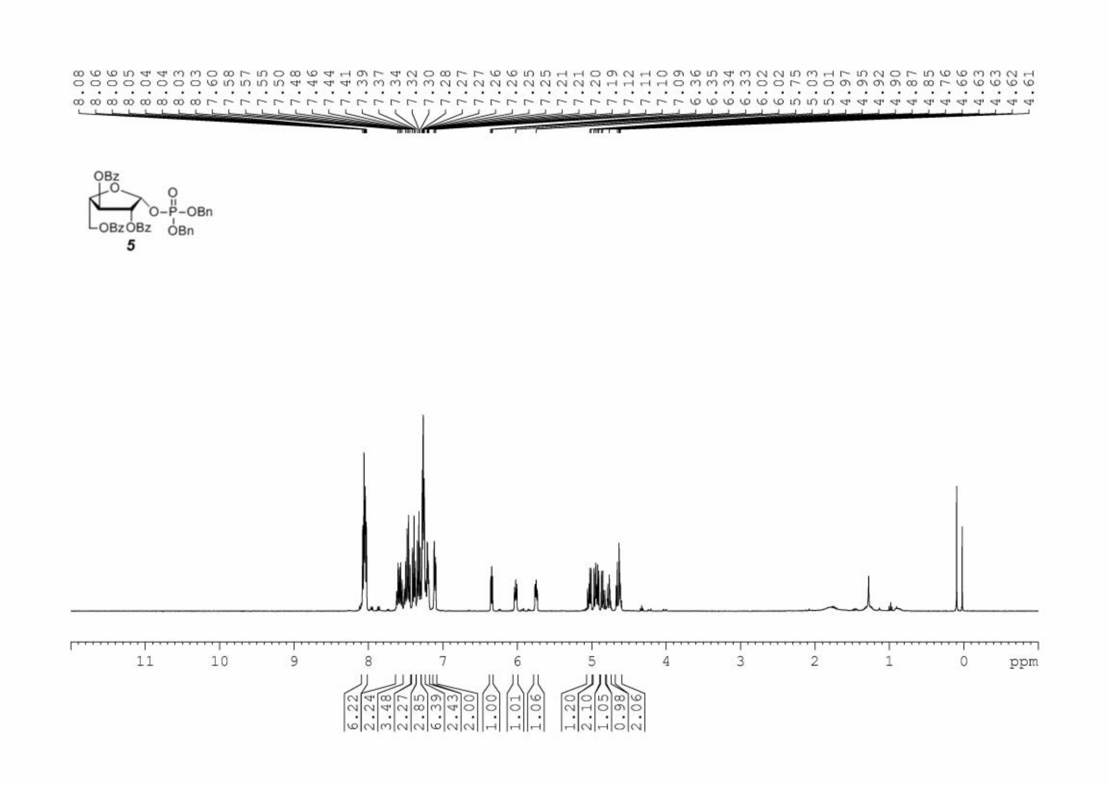

Figure S1. ^{1}H NMR of **5**

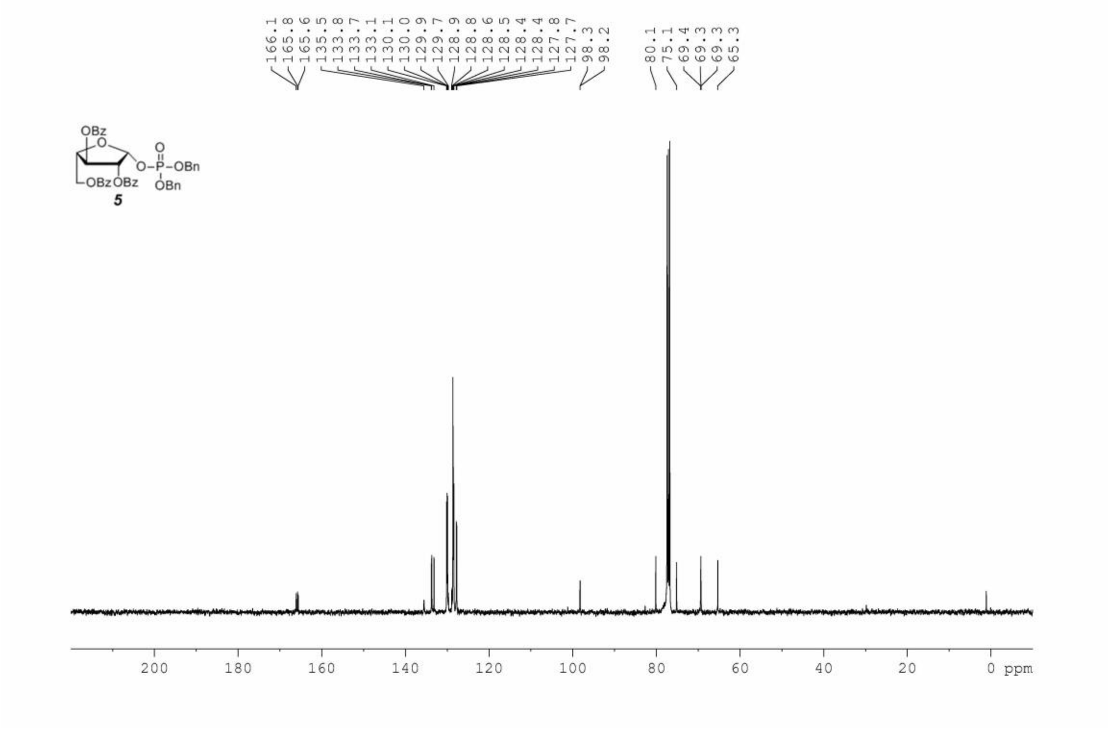

Figure S2. ^{13}C NMR of **5**

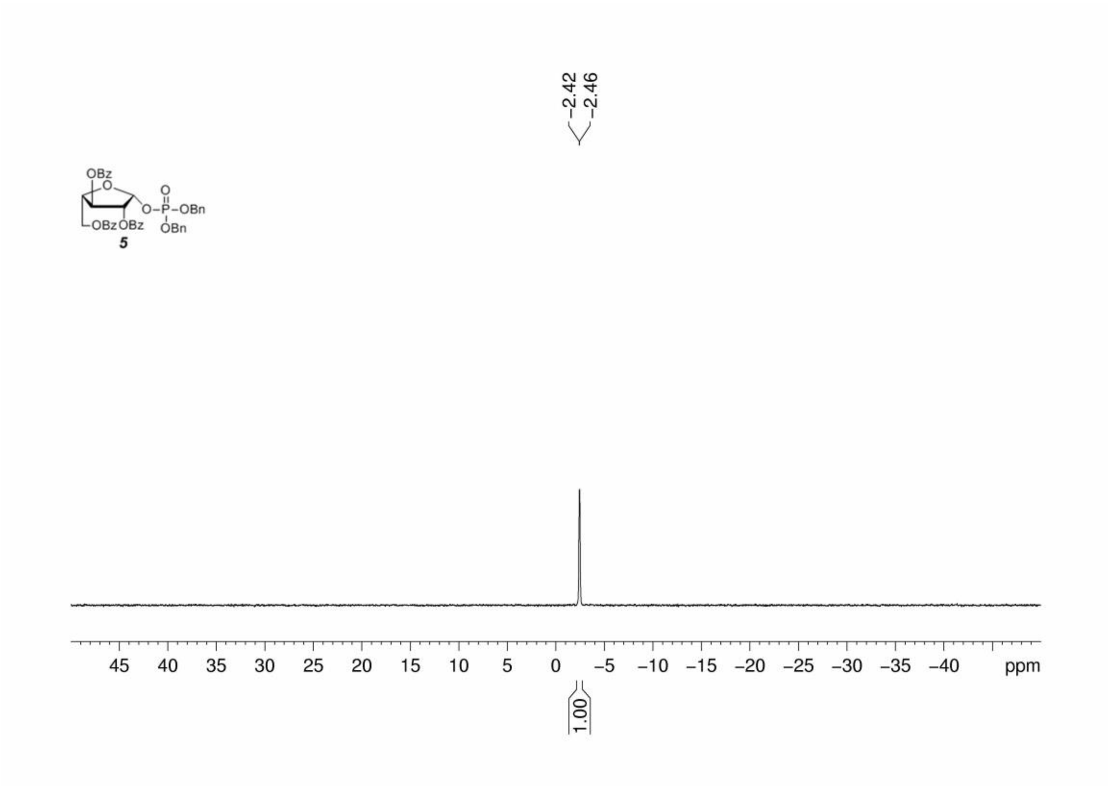

Figure S3. ^{31}P NMR of **5**

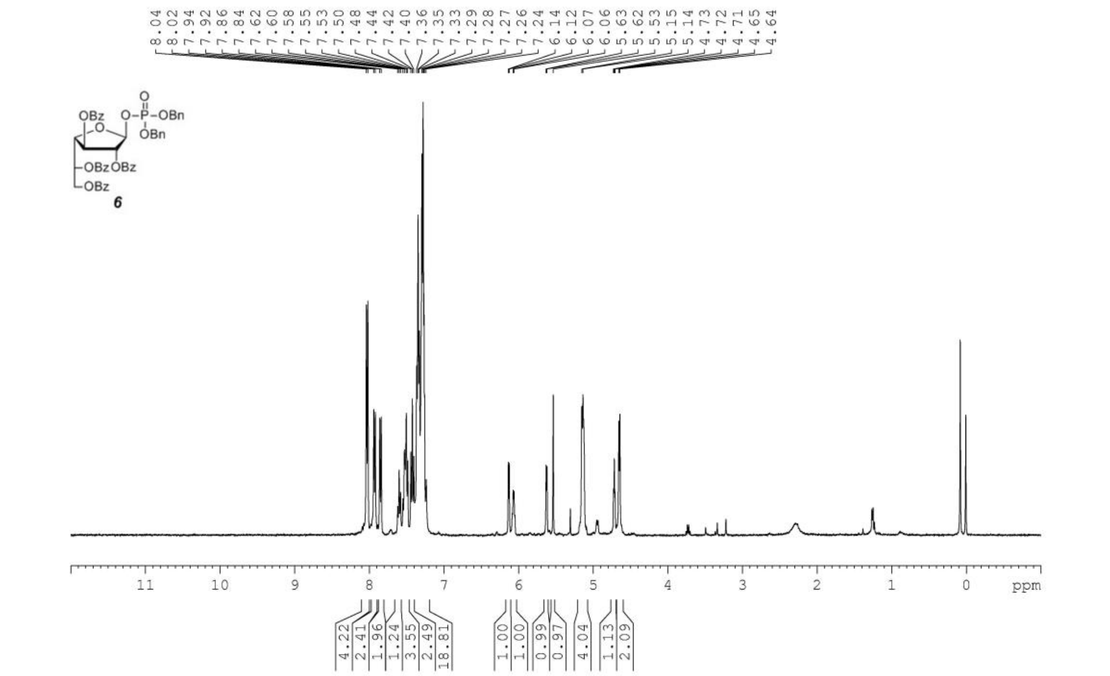

Figure S4. ^{1}H NMR of **6**

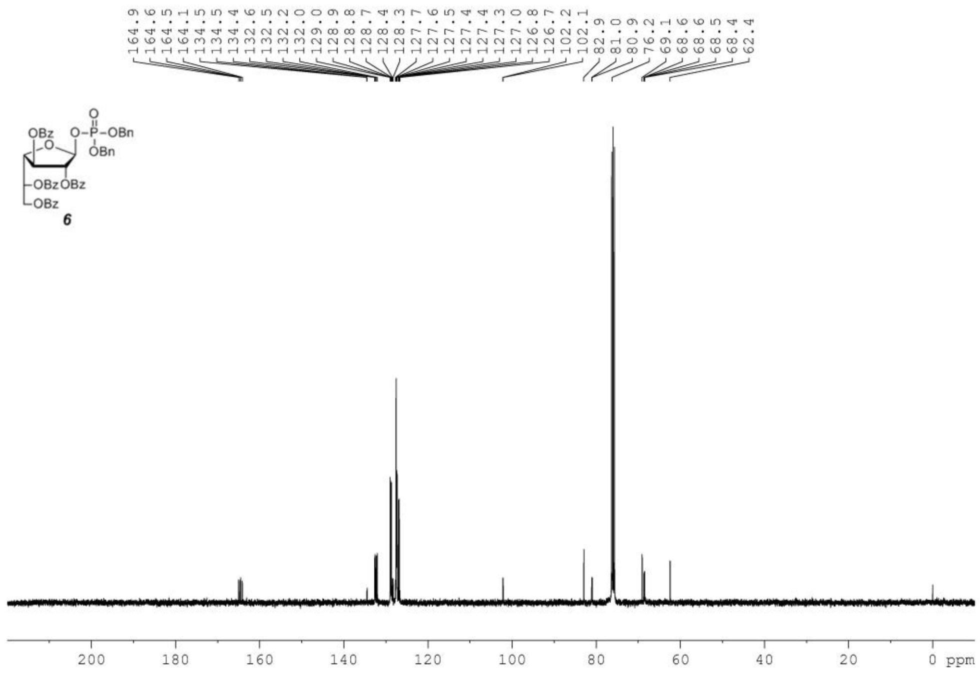

Figure S5. ^{13}C NMR of **6**

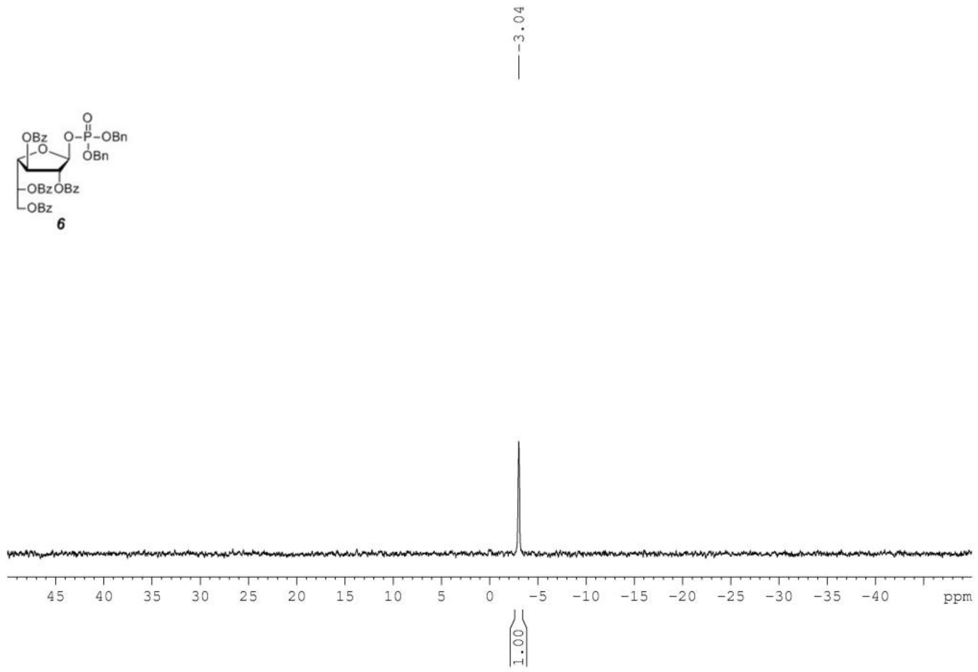

Figure S6. ^{31}P NMR of **6**

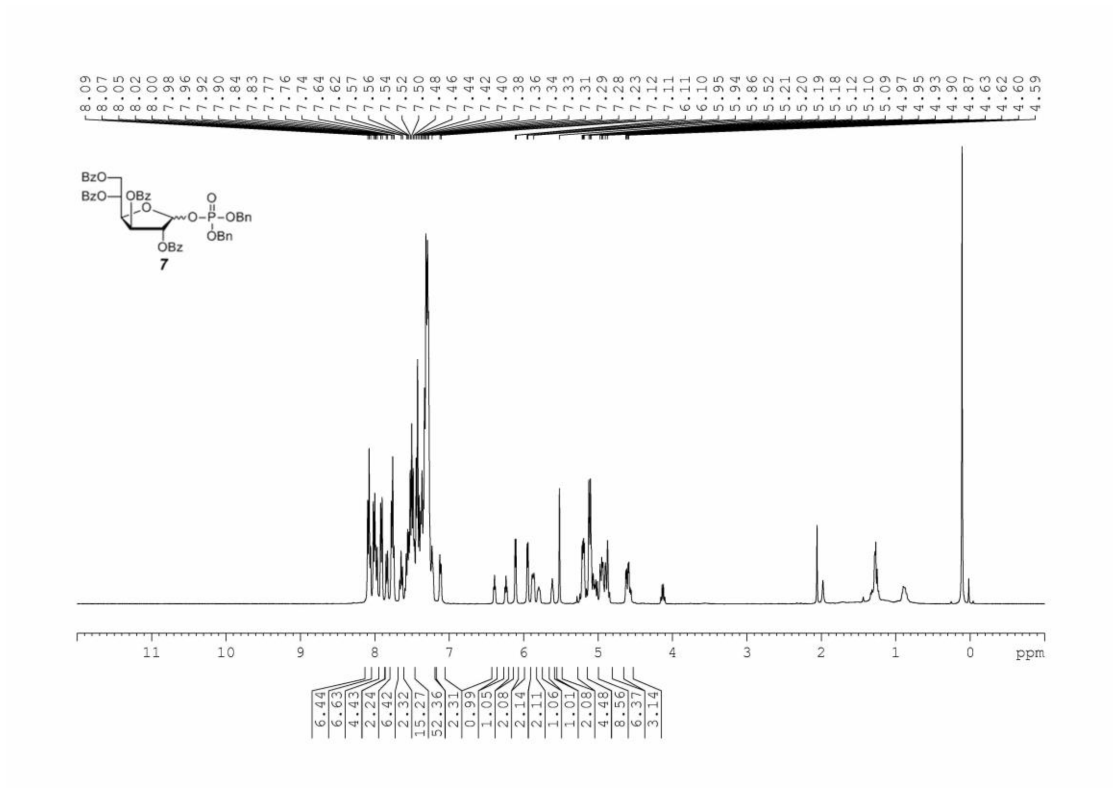

Figure S7. ^{1}H NMR of **7**

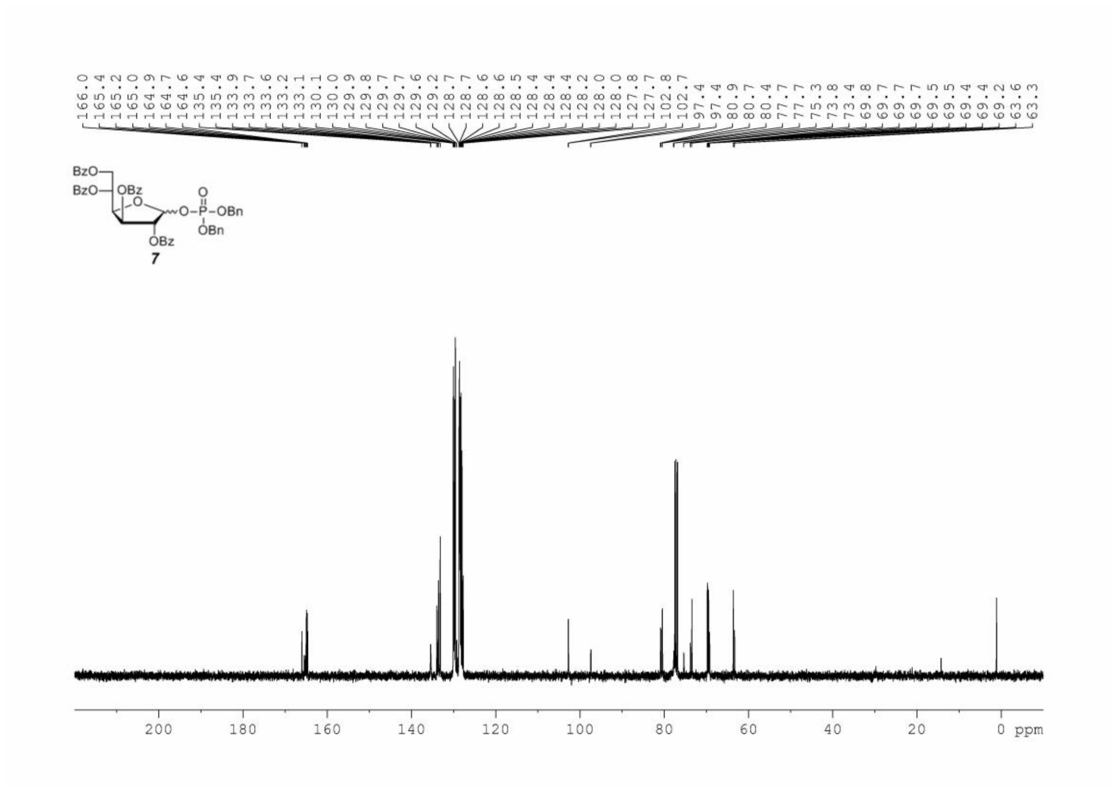

Figure S8. ^{13}C NMR of **7**

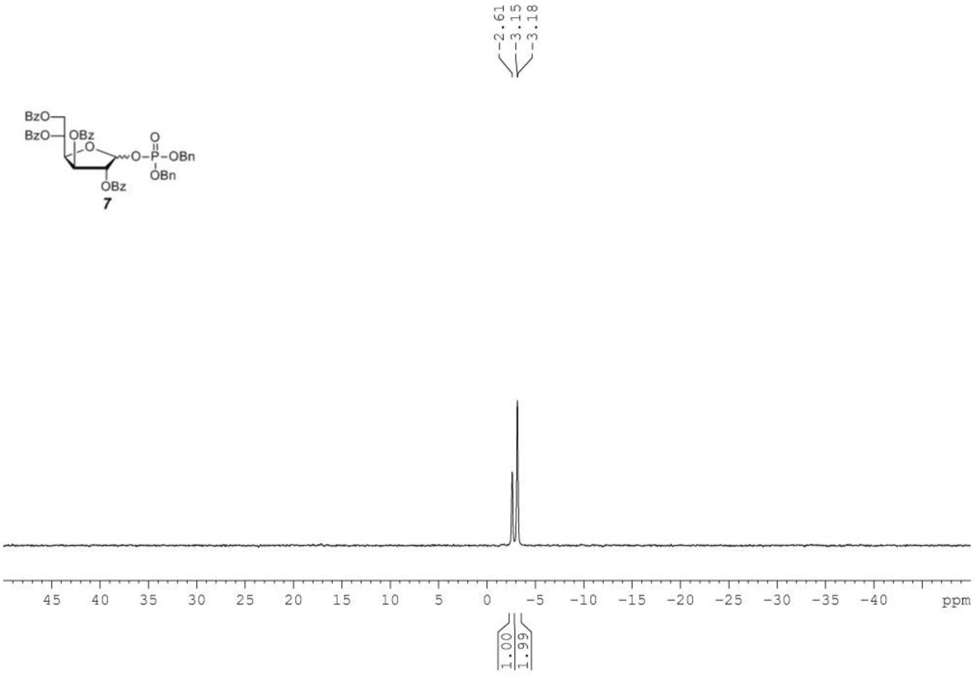

Figure S9. ^{31}P NMR of **7**

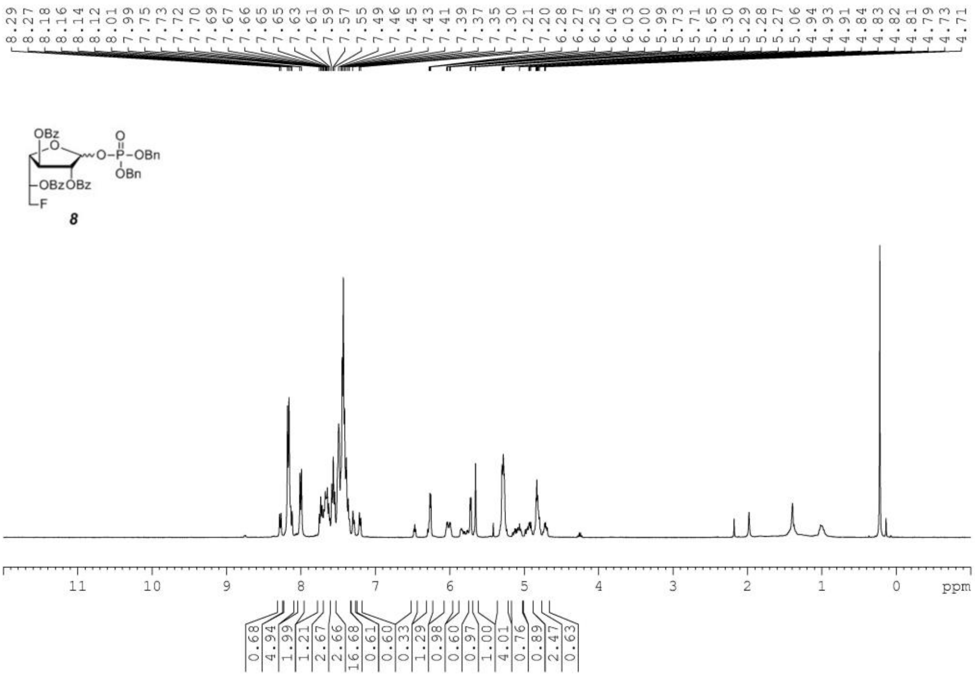

Figure S10. ^{1}H NMR of **8**

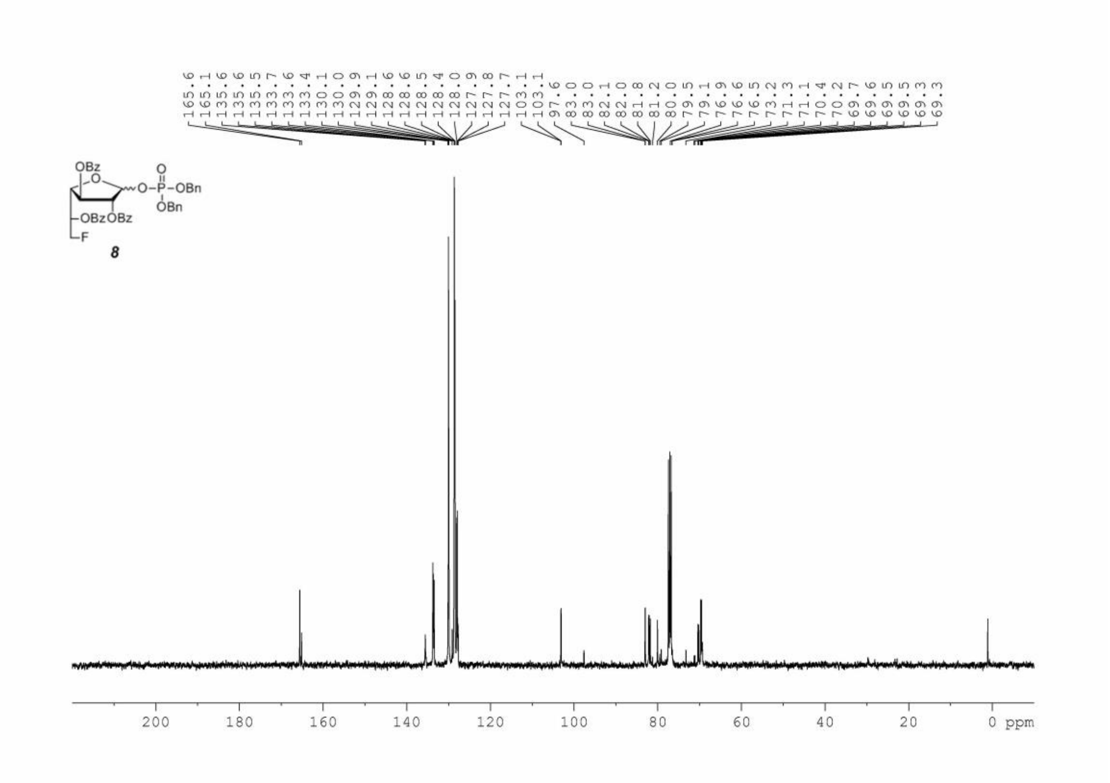

Figure S11. ^{13}C NMR of **8**

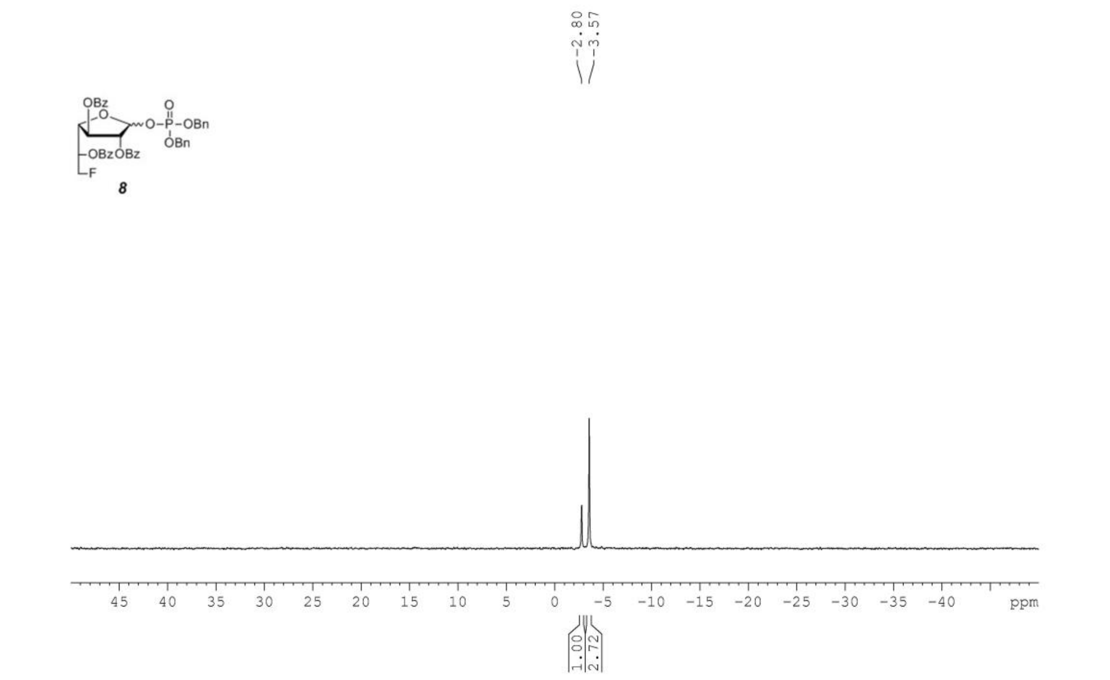

Figure S12. ^{31}P NMR of **8**

2. The NMR spectra of 9–13

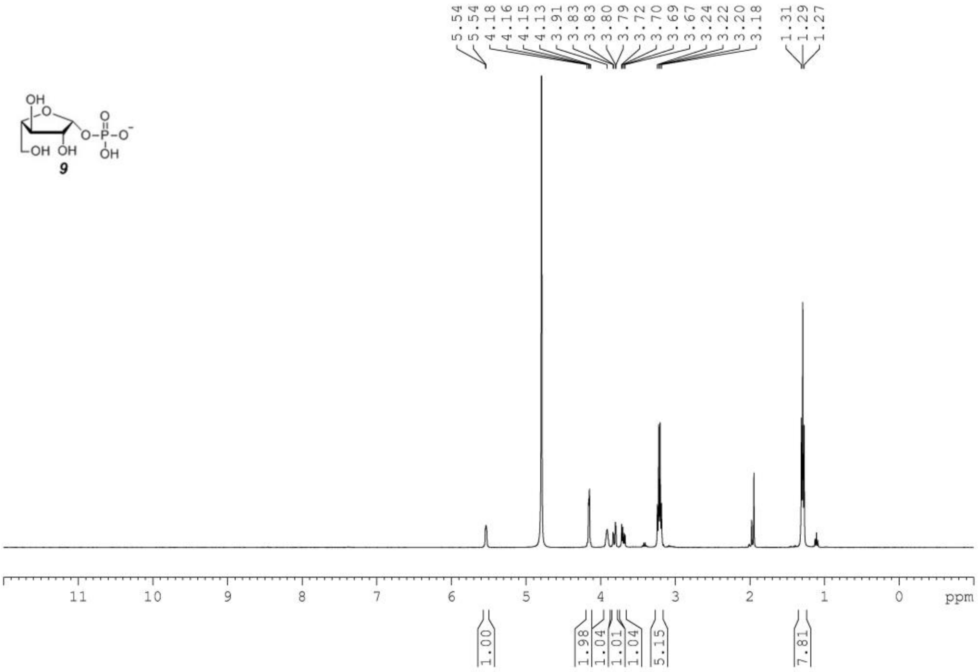

Figure S13. ^{1}H NMR of **9**

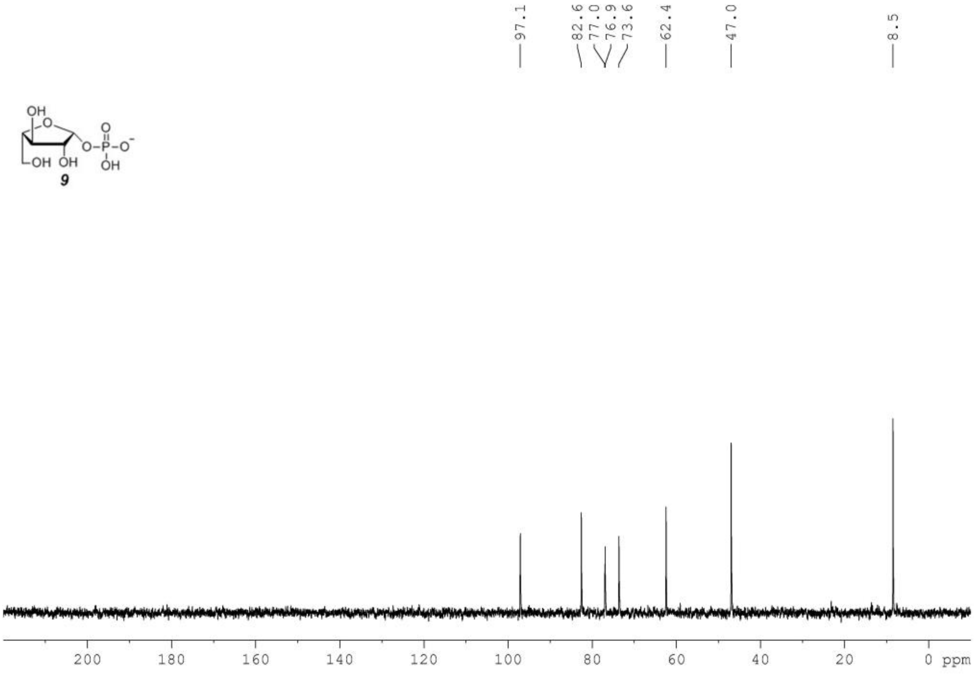

Figure S14. ^{13}C NMR of **9**

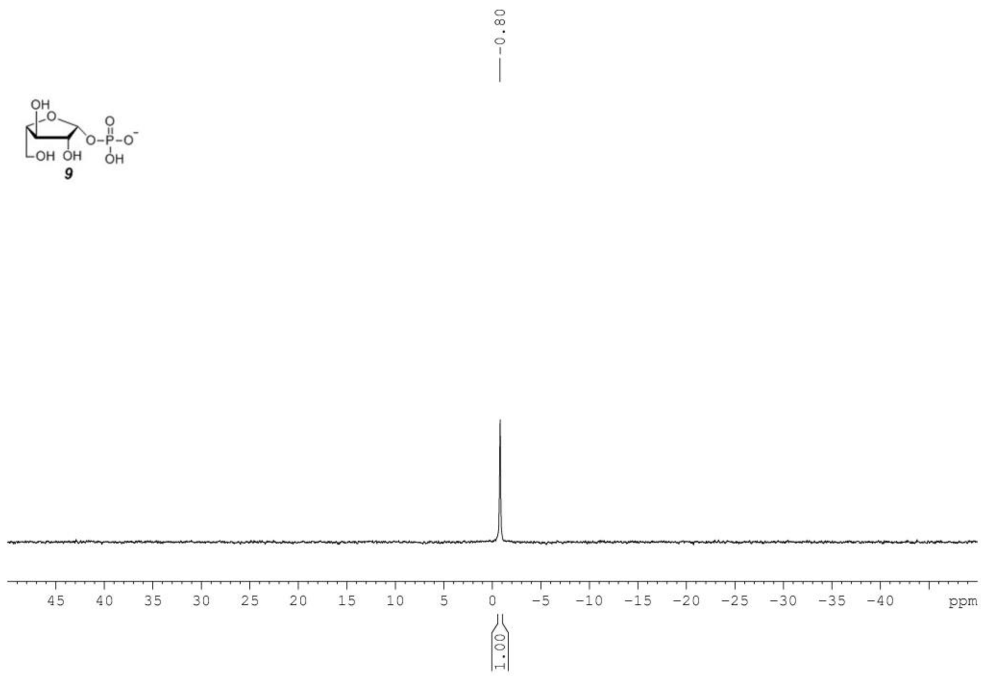

Figure S15. ^{31}P NMR of **9**

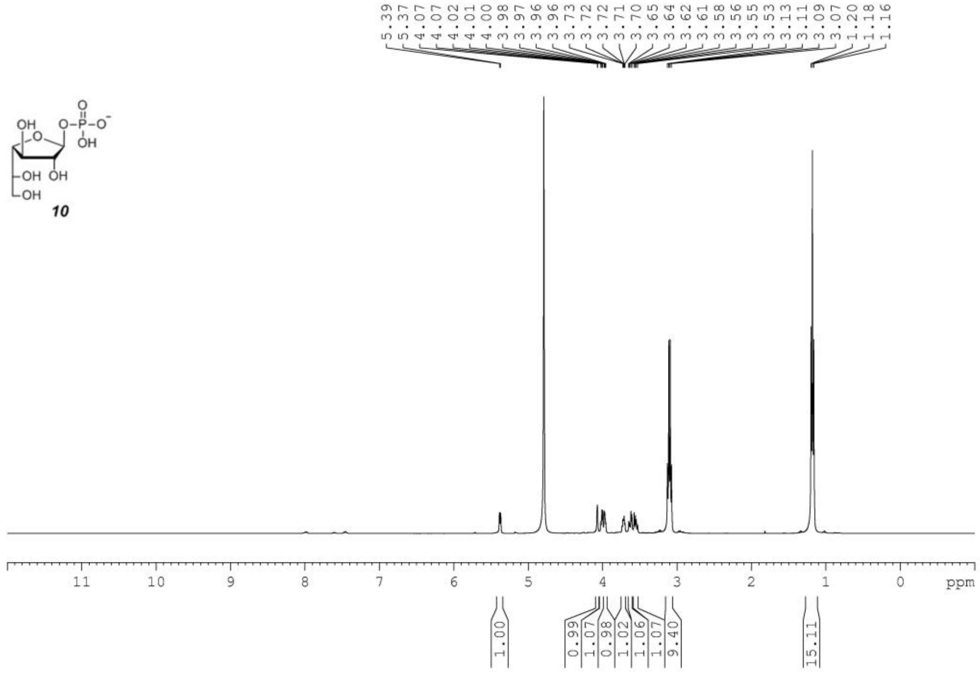

Figure S16. ^{1}H NMR of **10**

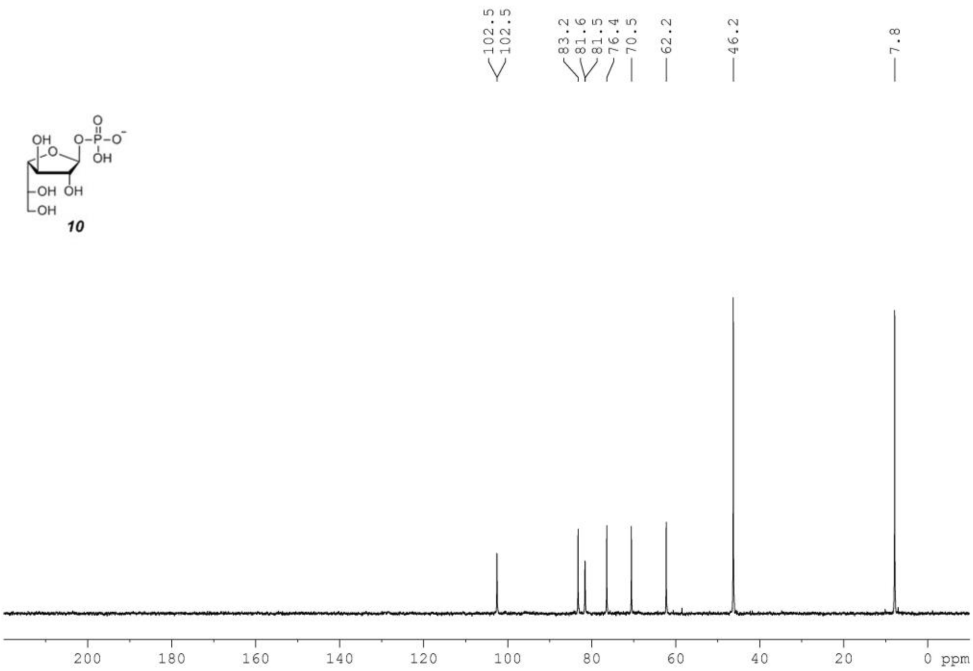

Figure S17. ^{13}C NMR of **10**

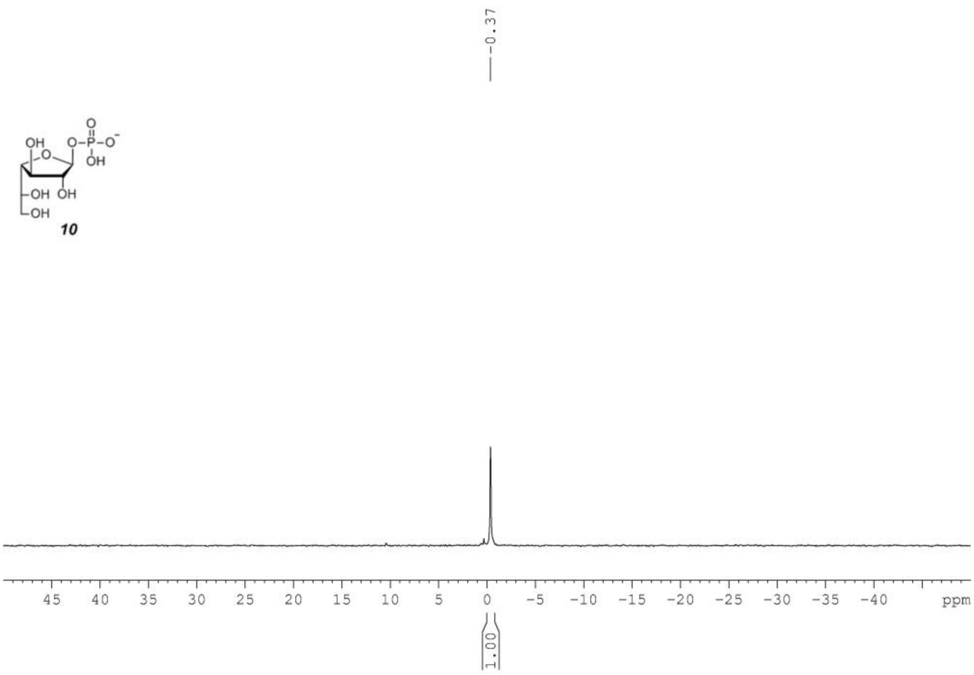

Figure S18. ^{31}P NMR of **10**

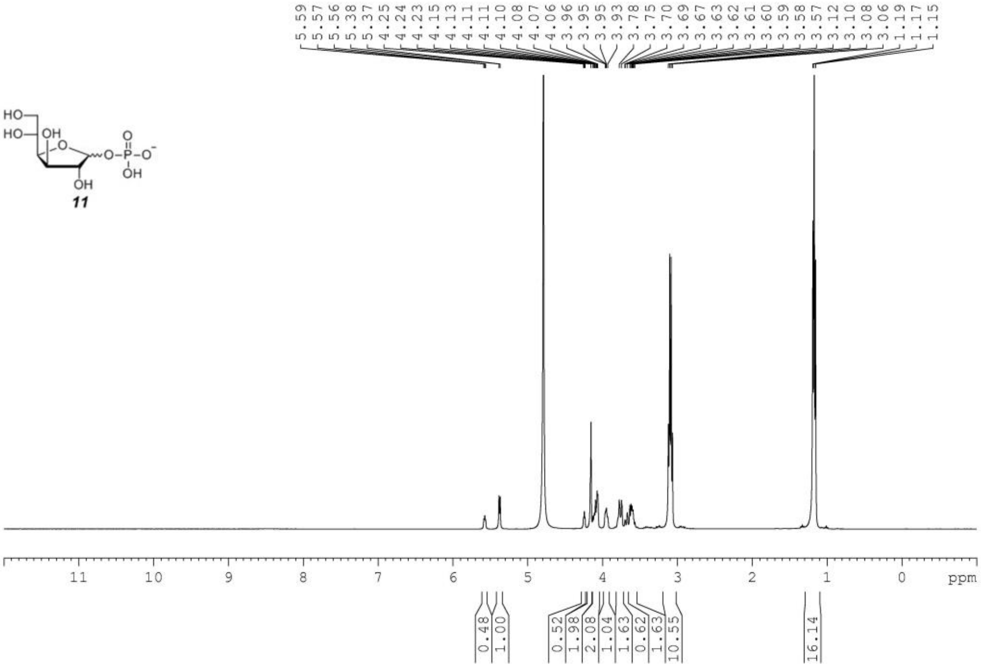

Figure S19. ^{1}H NMR of **11**

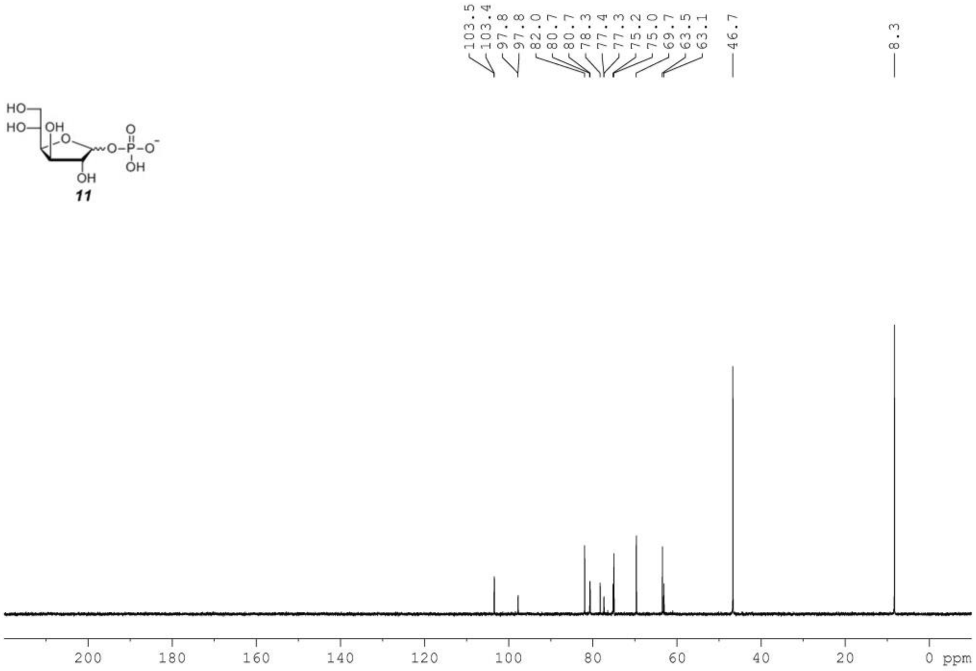

Figure S20. ^{13}C NMR of **11**

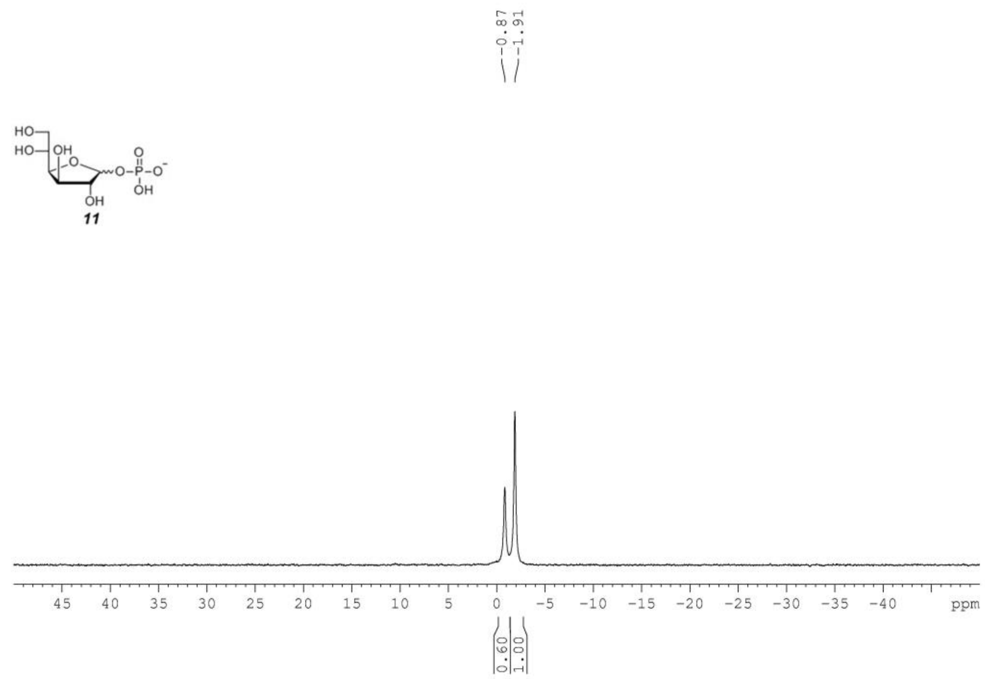

Figure S21. ^{31}P NMR of **11**

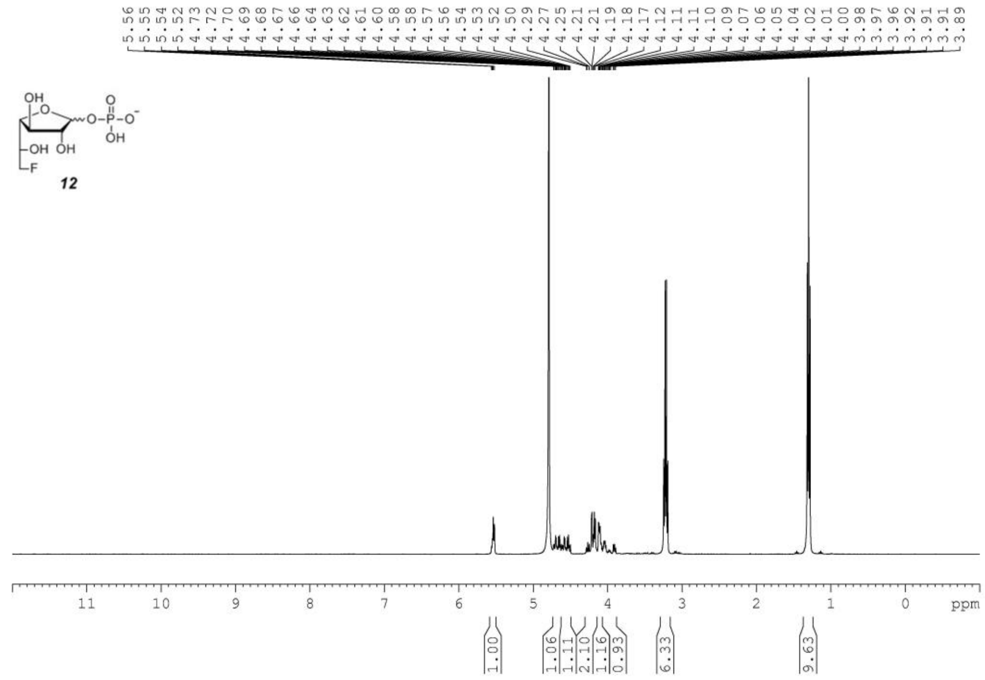

Figure S22. ^{1}H NMR of **12**

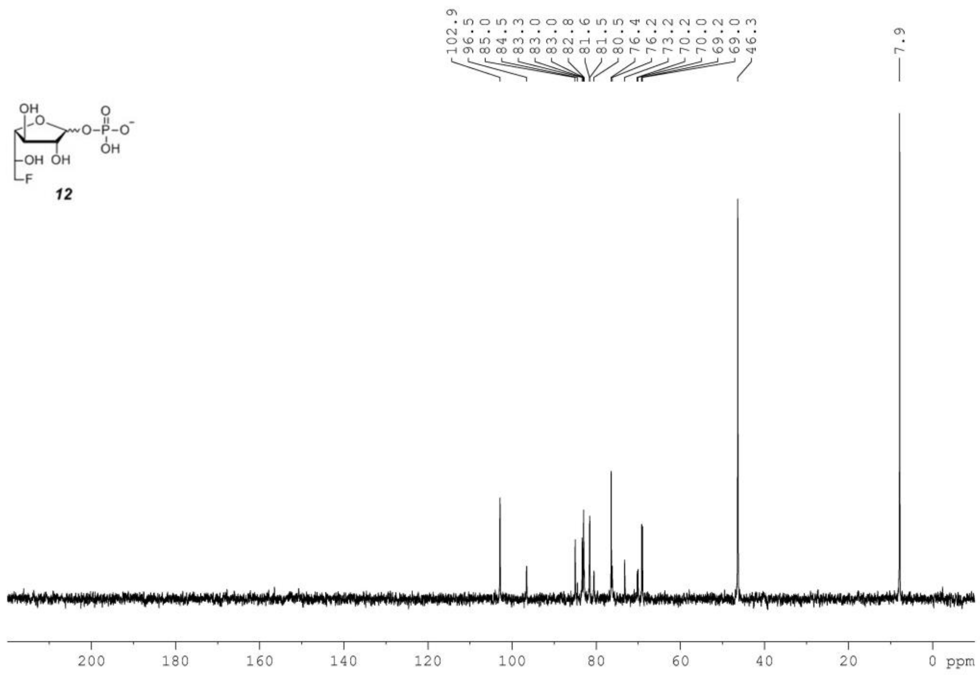

Figure S23. ^{13}C NMR of **12**

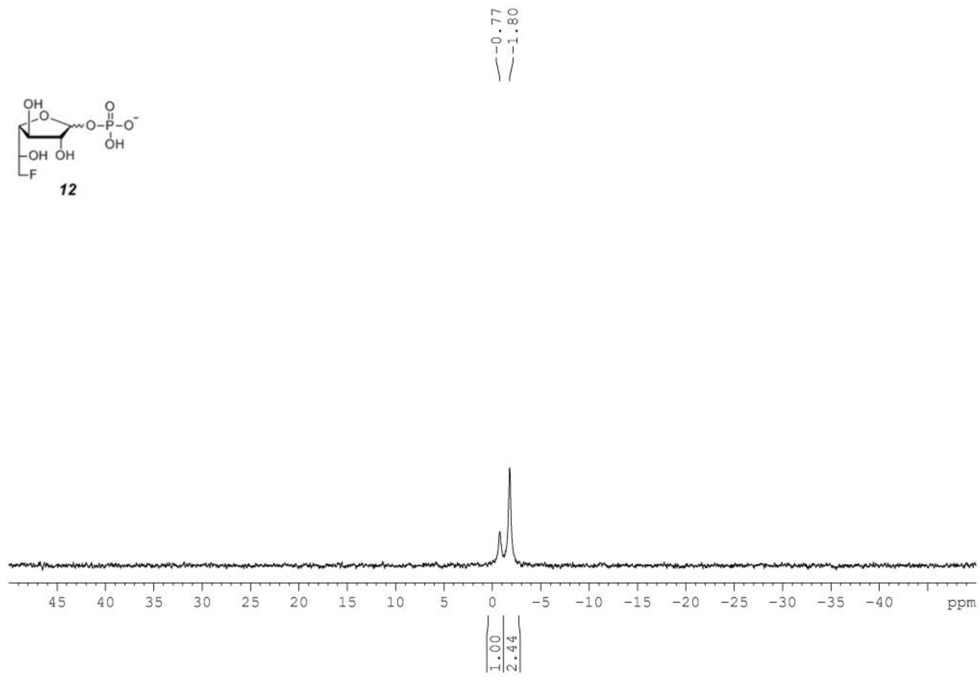

Figure S24. ^{31}P NMR of **12**

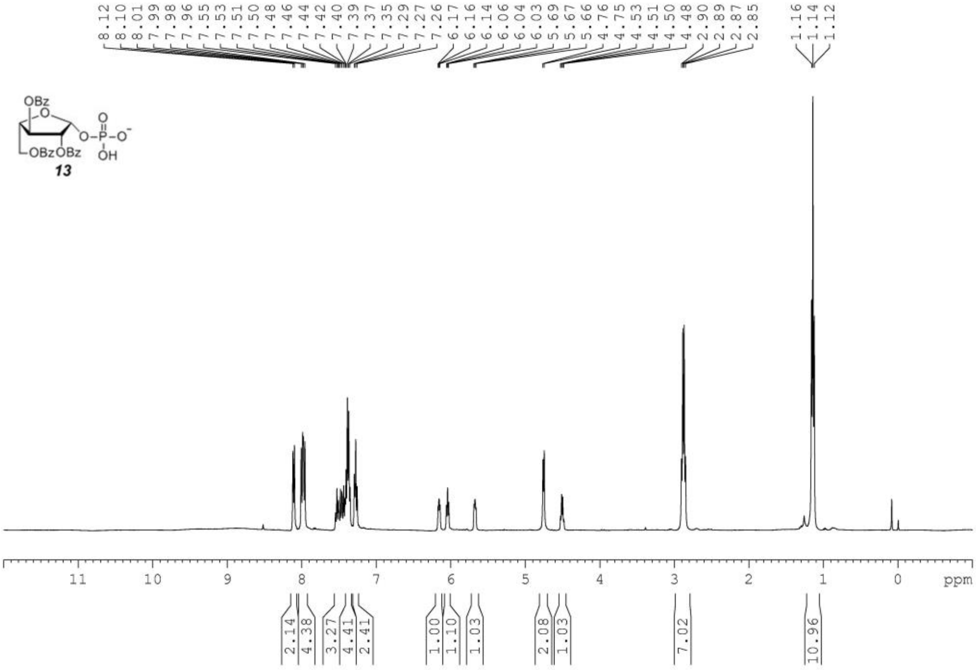

Figure S25. ^{1}H NMR of **13**

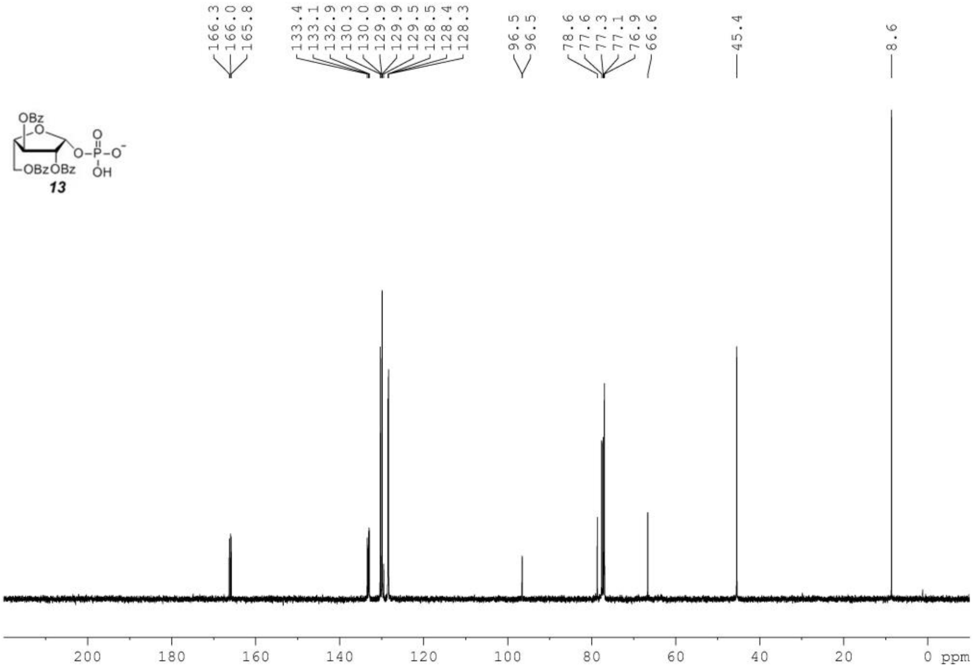

Figure S26. ^{13}C NMR of **13**

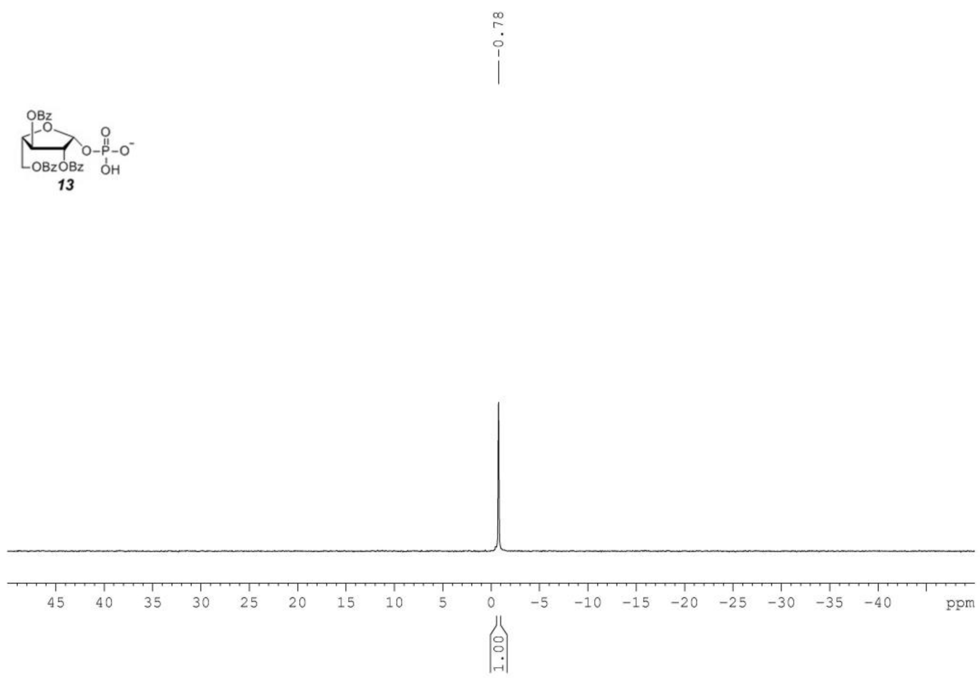

Figure S27. ^{31}P NMR of **13**

3. The NMR spectra of 15–19

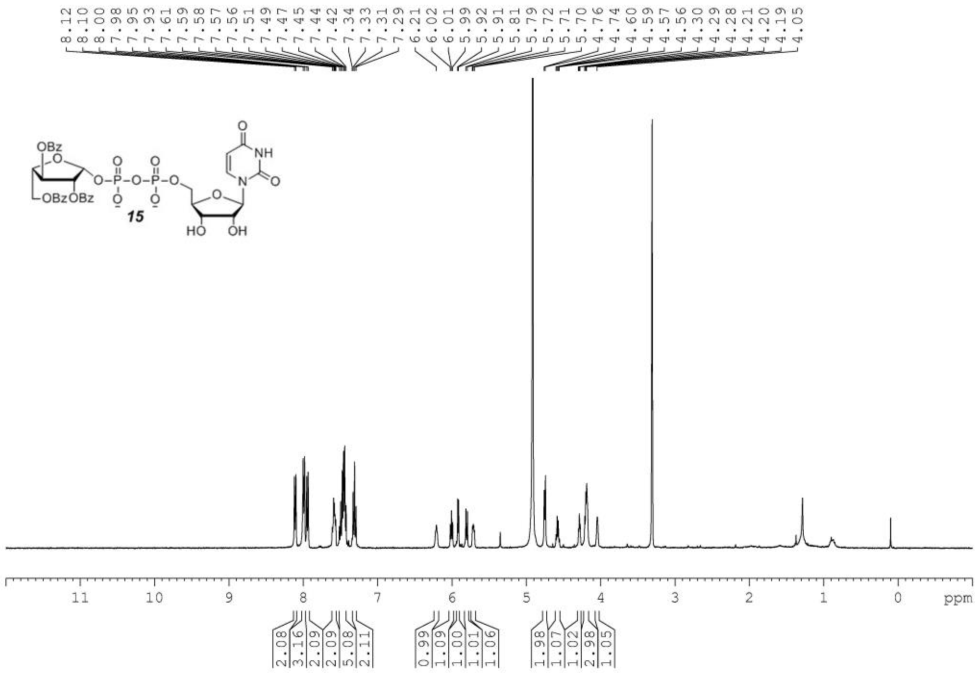

Figure S28. ^{1}H NMR of **15**

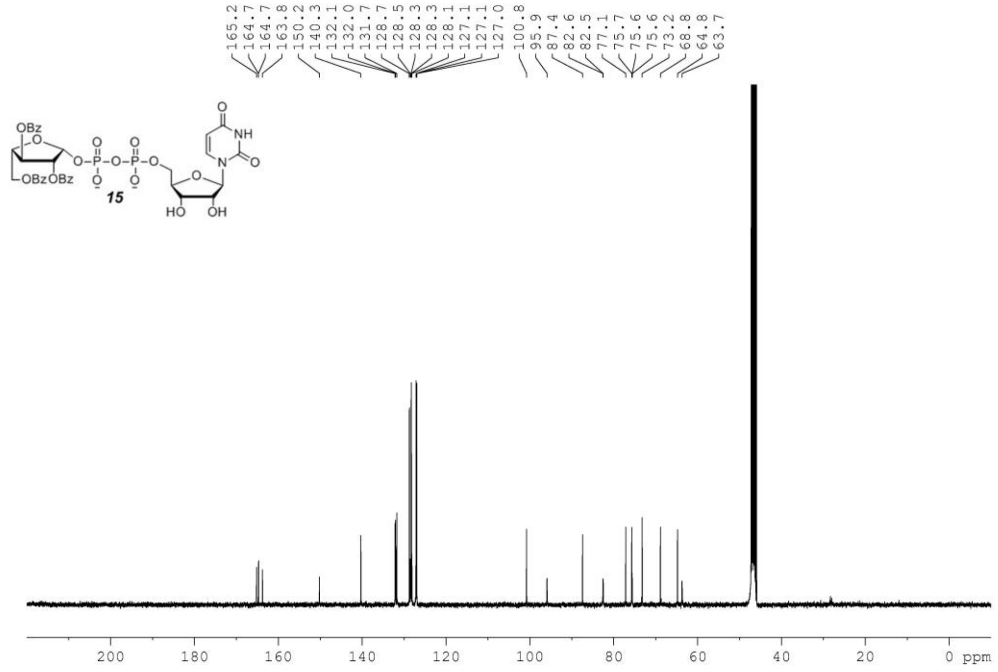

Figure S29. ^{13}C NMR of **15**

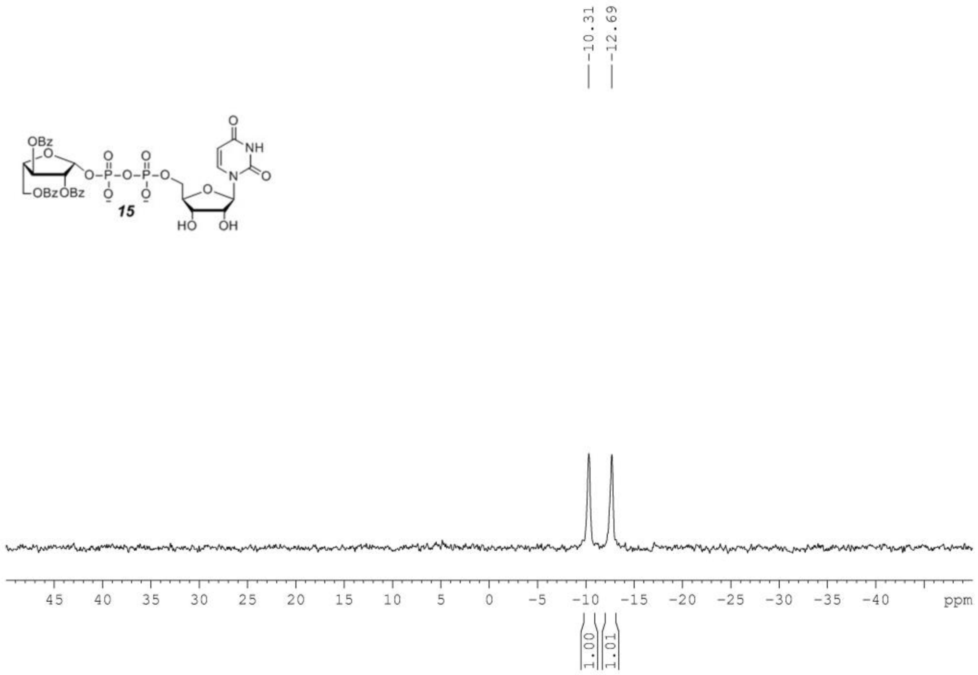

Figure S30. ^{31}P NMR of **15**

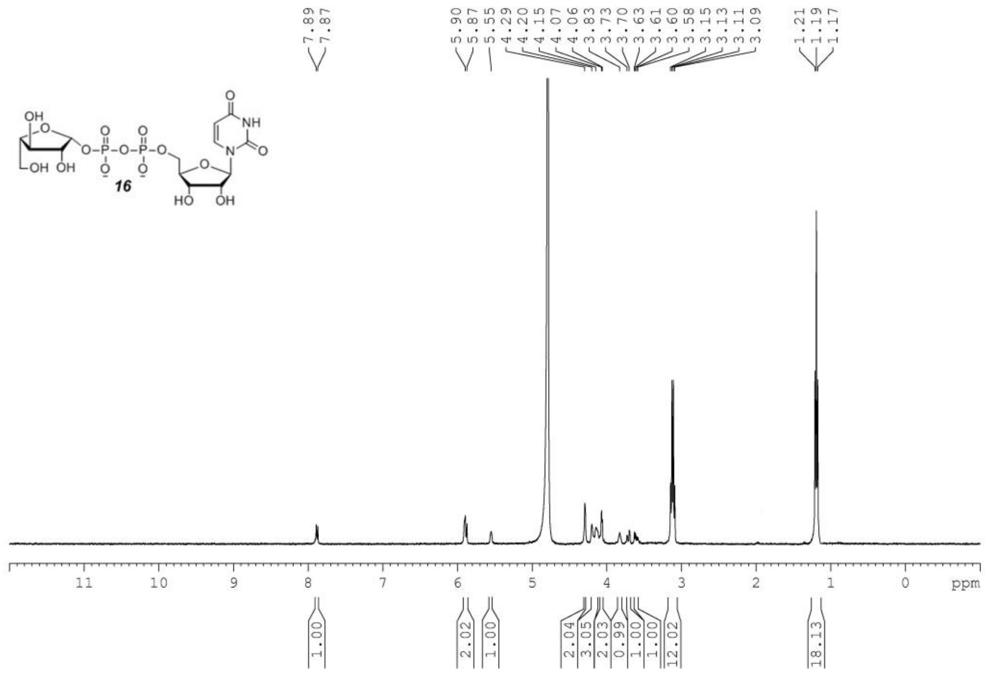

Figure S31. ^{1}H NMR of **16**

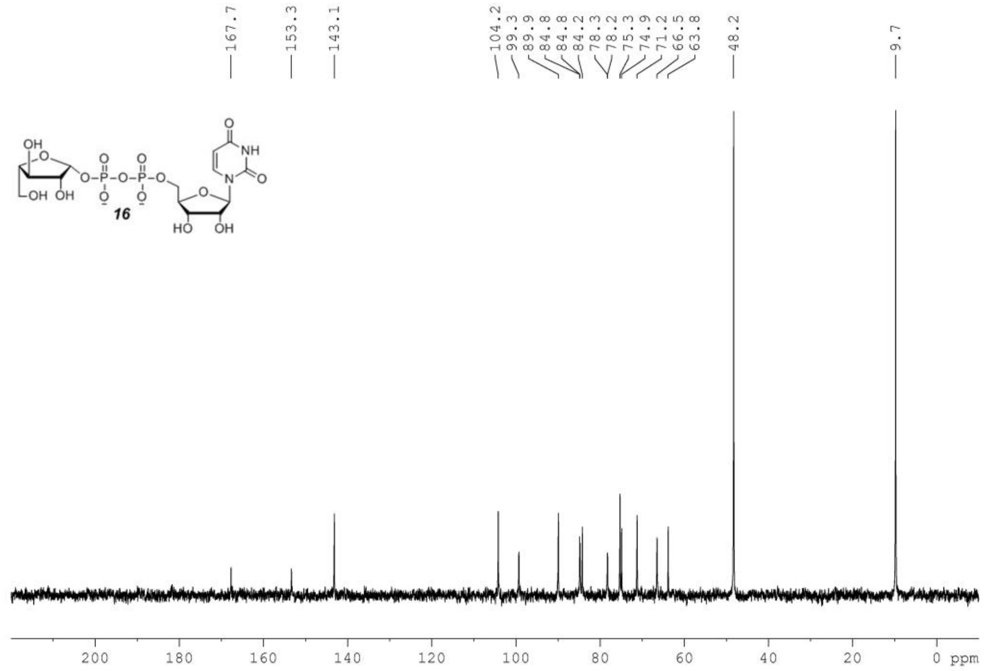

Figure S32. ^{13}C NMR of **16**

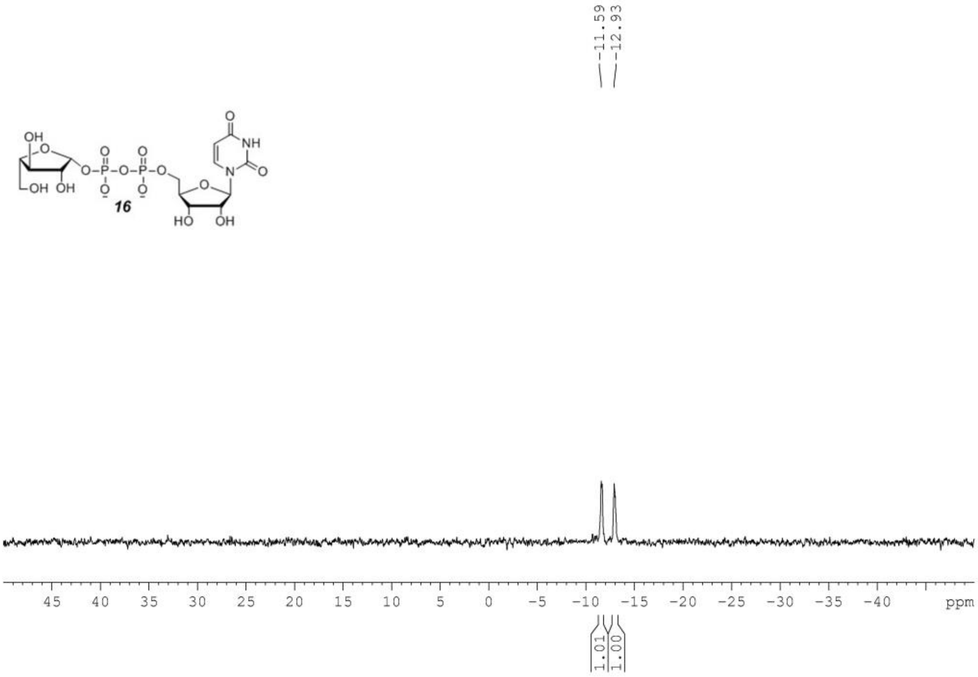

Figure S33. ^{31}P NMR of **16**

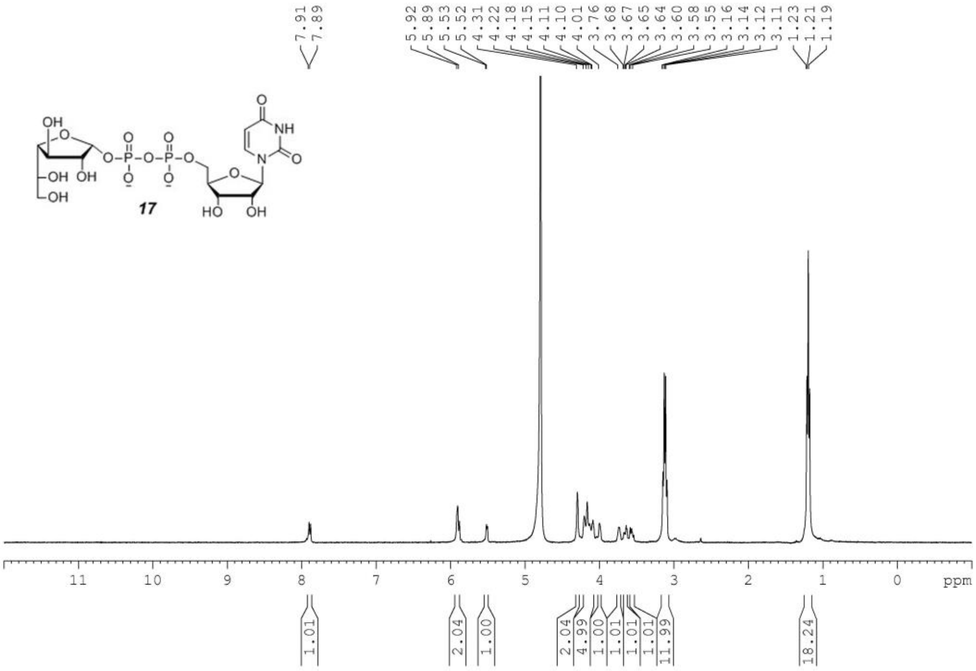

Figure S34. ^{1}H NMR of **17**

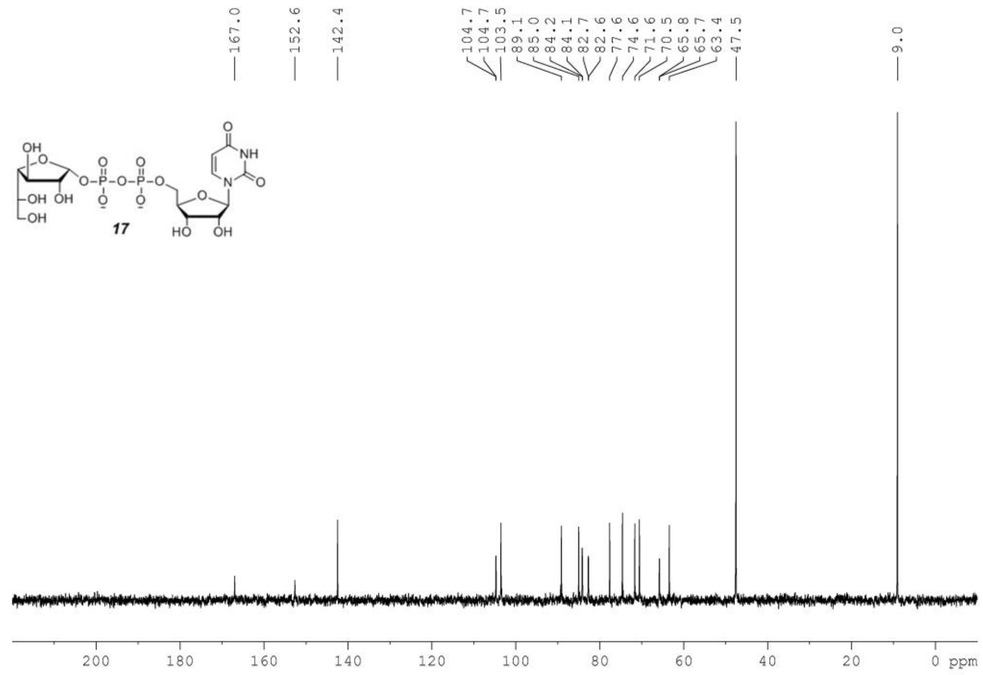

Figure S35. ^{13}C NMR of **17**

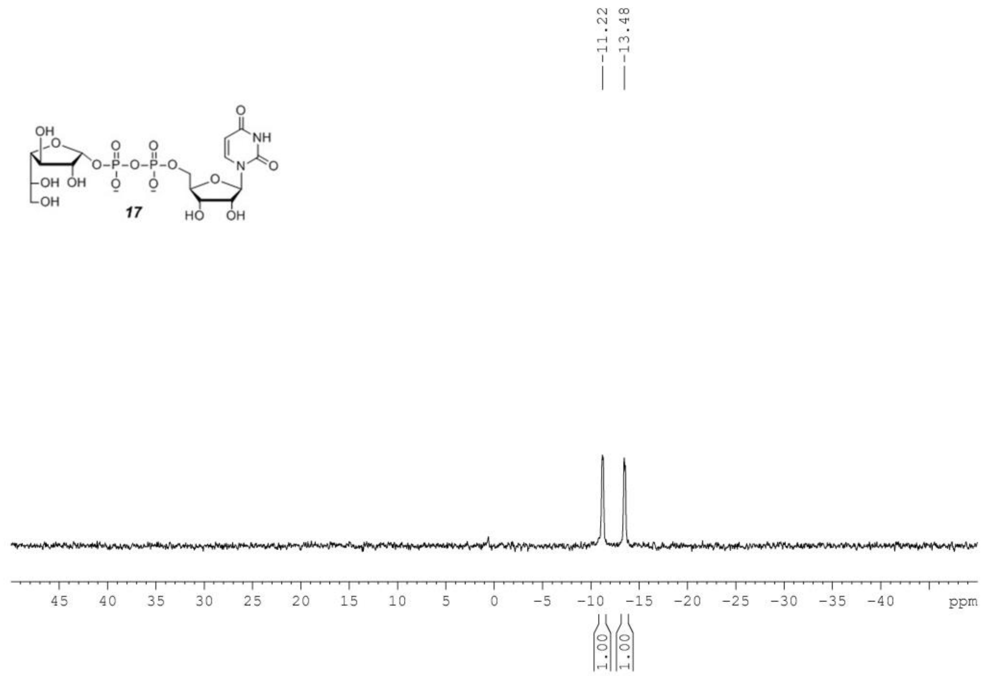

Figure S36. ^{31}P NMR of **17**

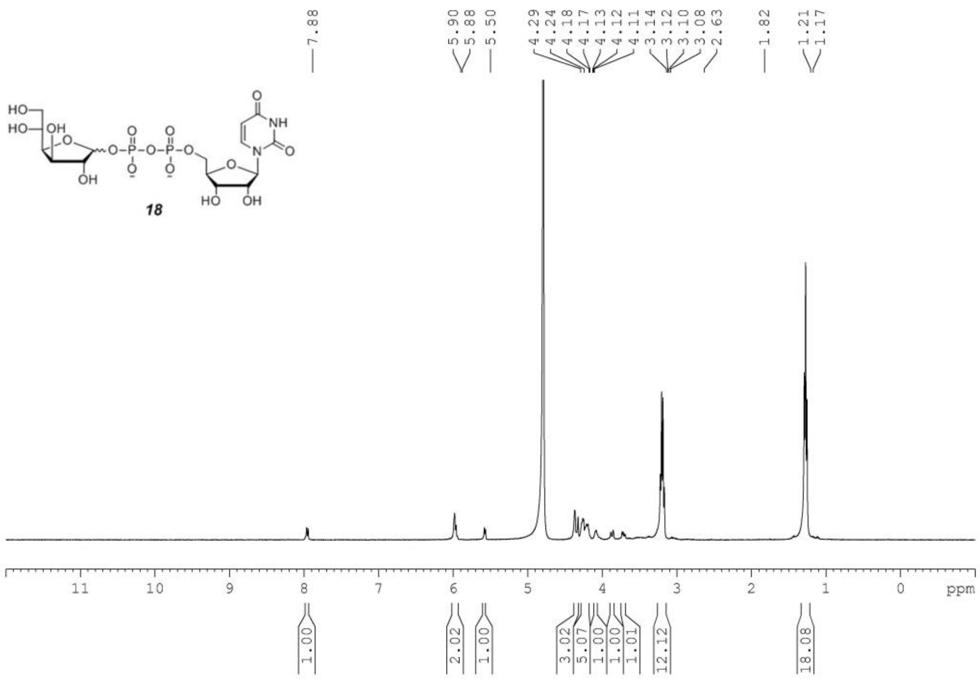

Figure S37. ^{1}H NMR of **18**

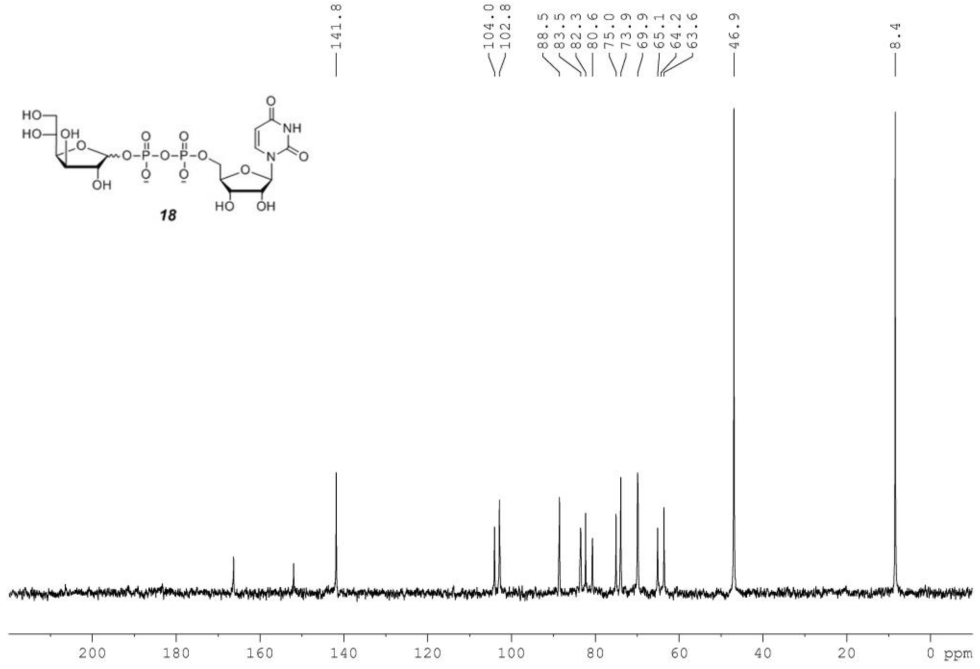

Figure S38. ^{13}C NMR of **18**

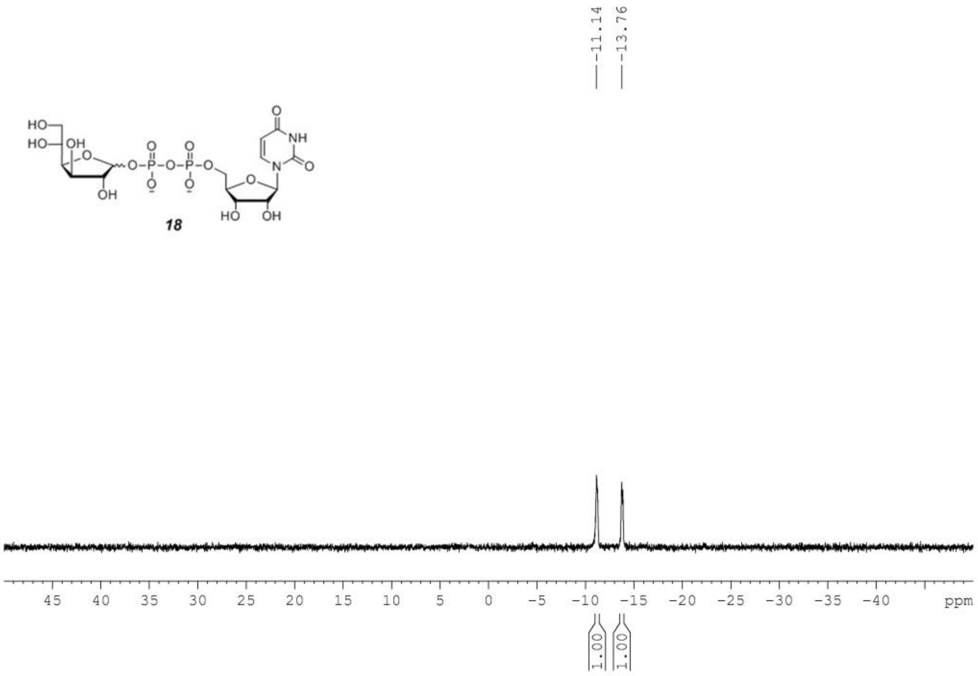

Figure S39. ^{31}P NMR of **18**

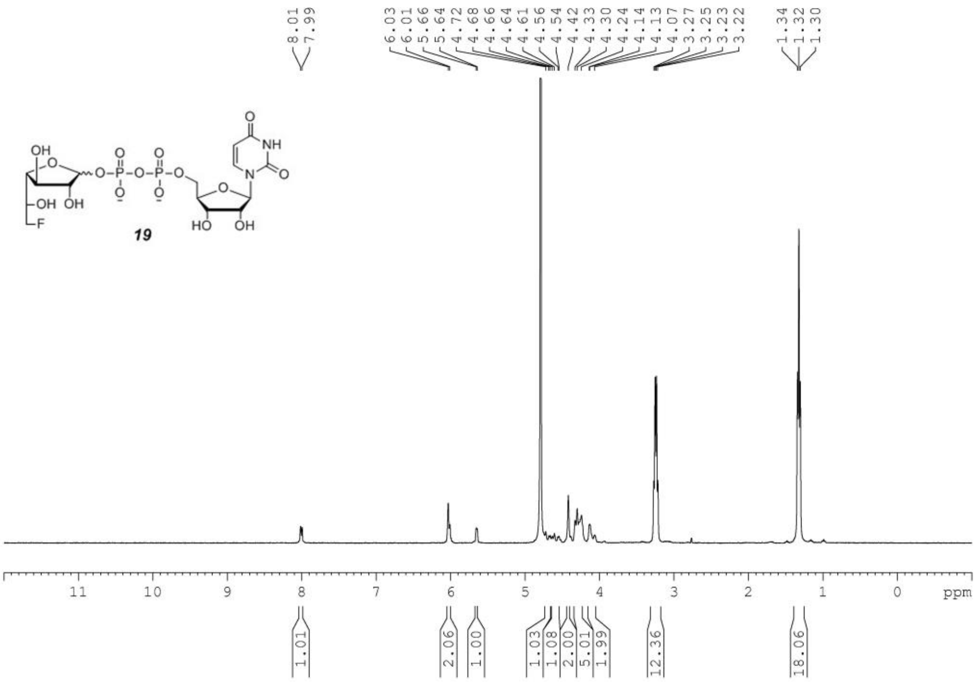

Figure S40. ^{1}H NMR of **19**

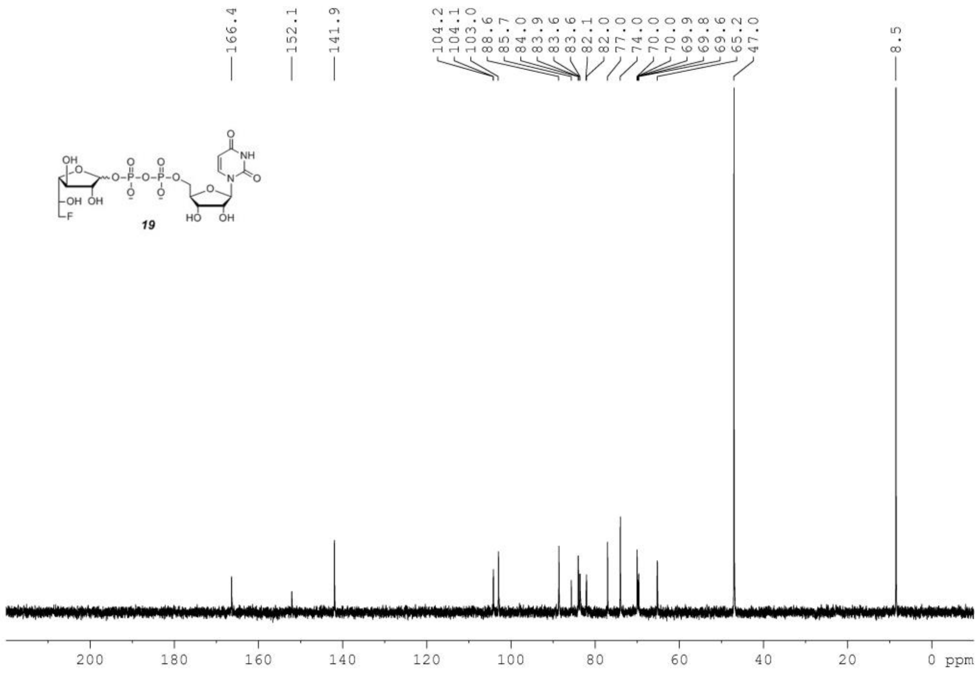

Figure S41. ^{13}C NMR of **19**

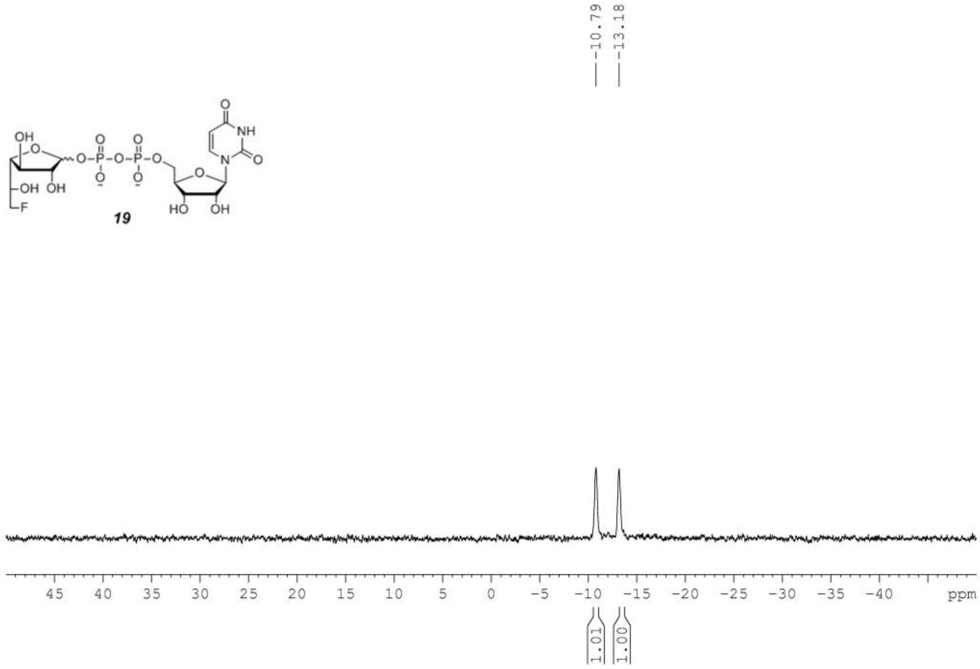

Figure S42. ^{31}P NMR of **19**

4. The HPLC traces of 16–19

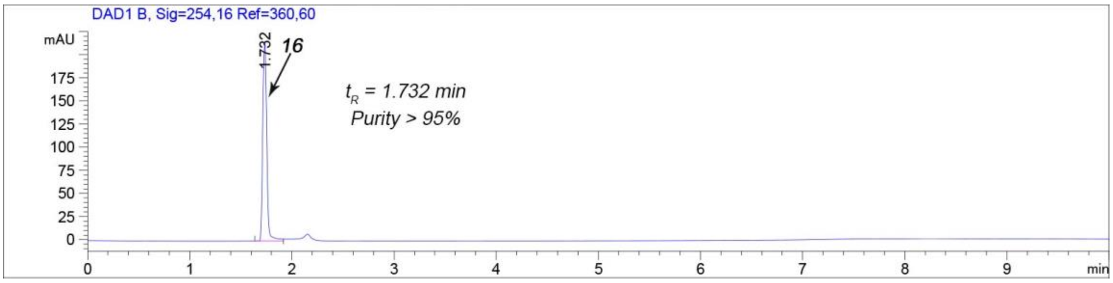

Figure S43. HPLC trace of **16**

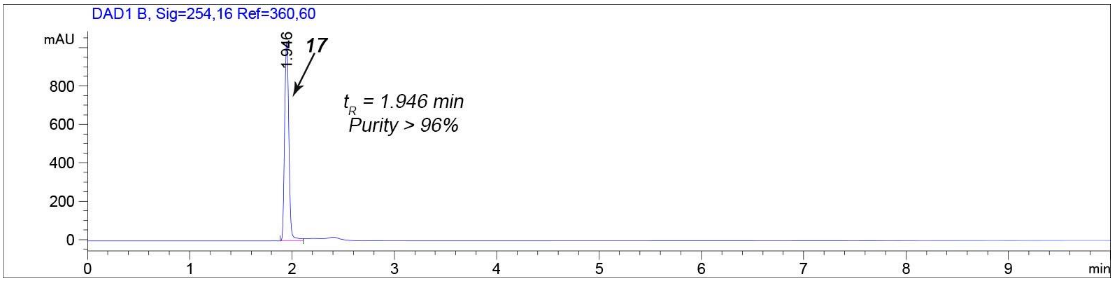

Figure S44. HPLC trace of **17**

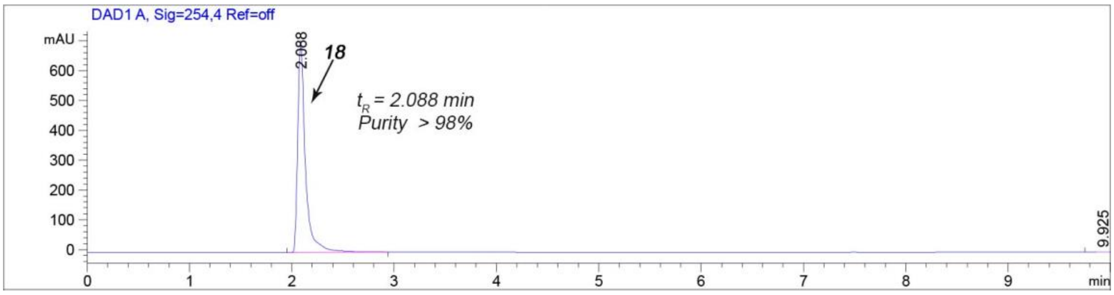

Figure S45. HPLC trace of **18**

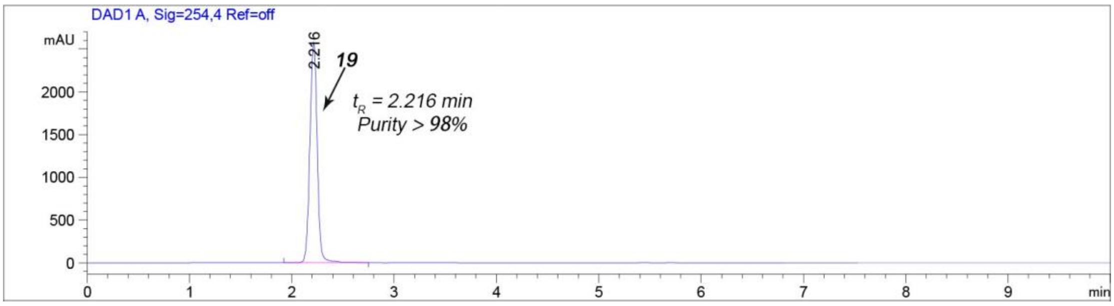

Figure S46. HPLC trace of **19**
